# Gene Expression Analysis Links Autocrine Vasoactive Intestinal Peptide and ZEB1 in Gastrointestinal Cancers

**DOI:** 10.3390/cancers15133284

**Published:** 2023-06-22

**Authors:** Ishani H. Rao, Edmund K. Waller, Rohan K. Dhamsania, Sanjay Chandrasekaran

**Affiliations:** 1 Department of Hematology and Medical Oncology, Emory University School of Medicine, Atlanta, GA 30322, USA; 2 Philadelphia College of Osteopathic Medicine (PCOM)-Georgia Campus, Suwanee, GA 30024, USA; 3 Harold C. Simmons Cancer Center, University of Texas Southwestern Medical Center, Dallas, TX 75390, USA

**Keywords:** VIP, ZEB1, cancer, EMT, gastrointestinal cancers

## Abstract

**Simple Summary:**

The downstream signaling mechanisms and importance of the autocrine secretion of the vasoactive intestinal peptide (VIP) in cancer remains poorly understood. We hypothesized that VIP expression may promote cancer-associated signaling pathways. We analyzed gene sequencing data from cancer and healthy tissues based on the co-expression data of the VIP with 760 cancer-related genes. We identified a meaningful and novel association between the VIP and transcription factor ZEB1 in healthy and malignant human gastrointestinal tissues. ZEB1 is a known regulator of cancer EMT (epithelial–mesenchymal transition). Gene set analysis further supports the overlap in the EMT and cell cycle pathways. Our results identify a potentially novel function of the autocrine VIP as an important signaling peptide and biomarker of ZEB1-mediated EMT.

**Abstract:**

VIP (vasoactive intestinal peptide) is a 28-amino acid peptide hormone expressed by cancer and the healthy nervous system, digestive tract, cardiovascular, and immune cell tissues. Many cancers express VIP and its surface receptors VPAC1 and VPAC2, but the role of autocrine VIP signaling in cancer as a targetable prognostic and predictive biomarker remains poorly understood. Therefore, we conducted an in silico gene expression analysis to study the mechanisms of autocrine VIP signaling in cancer. VIP expression from TCGA PANCAN tissue samples was analyzed against the expression levels of 760 cancer-associated genes. Of the 760 genes, 10 (MAPK3, ZEB1, TEK, NOS2, PTCH1 EIF4G1, GMPS, CDK2, RUVBL1, and TIMELESS) showed statistically meaningful associations with the VIP (Pearson’s *R*-coefficient > |0.3|; *p* < 0.05) across all cancer histologies. The strongest association with the VIP was for the epithelial–mesenchymal transition regulator ZEB1 in gastrointestinal malignancies. Similar positive correlations between the VIP and ZEB1 expression were also observed in healthy gastrointestinal tissues. Gene set analysis indicates the VIP is involved in the EMT and cell cycle pathways, and a high VIP and ZEB1 expression is associated with higher median estimate and stromal scores These findings uncover novel mechanisms for VIP- signaling in cancer and specifically suggest a role for VIP as a biomarker of ZEB1-mediated EMT. Further studies are warranted to characterize the specific mechanism of this interaction.

## 1. Introduction

Vasoactive Intestinal Peptide (VIP) is the final 28-amino acid (AA) product following multiple processing steps starting from prepro-VIP, a 170 AA precursor peptide [1,2] to which VIP primarily binds and signals through using two GPCRs (G-protein coupled receptors), VPAC1 and VPAC2 [3]. VIP, VPAC1, and VPAC2 are all broadly expressed in multiple healthy tissue types, in particular, tissues of the nervous system, digestive tract, and the respiratory, cardiovascular, and immune systems [4,5], and physiologically, VPAC/VIP signaling regulates embryonal development and growth, neuronal and epithelial cell signaling, gut absorption and motility, endocrine-mediated glycemia and circadian rhythms, immune cell responses, and carcinogenesis [6,7]. In the immune system, the VIP functions as a Type 2 cytokine [8]. VPAC1 is constitutively expressed in various immune cell populations, including T lymphocytes, macrophages, and dendritic cells [1,9]. Studies using transgenic mice have demonstrated that Th2 CD4+ T cells up-regulate VIP expression upon antigen presentation, while Th1 CD4+ T cells do not [10].

The importance of autocrine VIP as a biomarker or therapeutic target in cancer has remained elusive for many years. An in vitro study of >400 human primary tumors, tumor metastases, and normal tissues using subtype selective VPAC receptor autoradiography has established that most cancers express VPAC, with VPAC1 being the predominant receptor subtype in cancers and healthy tissues [11]. In clinical studies, radiolabeled-VIP binds VPAC and detects primary and metastatic tumors in a variety of cancer histologies [12,13], and serum VIP levels are > two-fold higher in patients with colon adenocarcinoma [14,15].

To date, the primary function of autocrine VIP signaling was thought to drive cancer cell proliferation. Mechanistically, VIP binds to surface VPAC1 and VPAC2 and generates downstream signals through adenylate cyclase to catalyze cyclic AMP (cAMP) synthesis and protein kinase A (PKA) activation [16]. The PKA phosphorylation of the CREB (cAMP-response element binding) transcription factor subsequently activates oncogenes such as c-Myc to drive cancer proliferation. In practice, in vitro studies with exogenous VIP or the pharmacologic inhibition of VIP signaling using VPAC1 and VPAC2 peptide antagonists have shown mixed results. No consistent increase in the proliferation rates of colon and pancreatic cancer cell lines was seen when adding exogenous VIP to cancer cells in vitro, and, in certain cell lines, exogenous VIP even inhibited growth [14]. Similarly, the pharmacologic inhibition of VIP signaling using VPAC1 and VPAC2 peptide antagonists in vitro demonstrated variable anti-tumor responses depending on cancer histology and the cell line tested, with positive responses primarily seen in pancreatic, colorectal, gastric, and breast cancer models [5,17,18,19,20,21]. Recent studies showed exogenous VIP feeds back to induce EGFR (epidermal growth factor receptor) and HER2 (human epidermal growth factor receptor 2) phosphorylation in breast cancer cells and inhibits the apoptotic effects of the RAS/RAF inhibitor sorafenib on cancer stem cells, suggesting there remain unknown downstream mechanisms of VIP [22,23,24,25].

The consistent co-expression of VIP and VPAC in cancer and the availability of radiotracers to follow the VIP/VPAC as predictive or prognostic biomarkers, coupled with the ability to inhibit VIP signaling via VPAC1 and VPAC2 peptide antagonists, supports the need to further characterize the VIP signaling landscape and determine its importance in the properties of cancer that have been described as the “hallmarks of cancer” [24,25]. In this exploratory analysis, we utilized an in silico gene expression model to uncover new downstream signaling pathways of VIP in cancer and corroborate these pathways in analyses of healthy tissues.

## 2. Materials and Methods

Gene association and expression: Cancer mRNA expression data for VIP and Pearson’s correlation (*R*) for VIP versus 760 other genes (gene panel based on the Nanostring nCounter^®^ Tumor Signaling 360™ profiling panel) were abstracted from the TCGA (The Cancer Genome Atlas) PANCAN dataset using the University of California Santa Cruz (UCSC) Xena platform and recorded into Microsoft Excel by cancer hallmark (per Nanostring designation) (Supplemental Table S1) [26,27,28]. The date of cutoff for data abstraction was 1 December 2020 and of the 12,839 samples available for analysis, 2036 null values were excluded (Supplemental Figure S1.1). Individual tissue sample mRNA expression levels and *R* coefficients for VIP and 10 identified lead genes were further abstracted for all TCGA tumor histologies. Histologies were also organized into sub-groups by the tissue germ layer of origin (ectoderm, endoderm, and mesoderm) (Supplemental Table S2). Healthy tissue gene expression data was obtained from the Genotype-Tissue Expression (GTEx) Project on 19 April 2023 (https://www.gtexportal.org/home/ (19 April 2023)) [29]. Cancer cell line-specific gene expression data was downloaded on 4/19/23 from the CCLE (cancer cell line encyclopedia) using the DepMap resource (Public Expression Dataset 22Q4) (https://depmap.org/portal (accessed on 19 April 2023)).

Statistical analysis: The strength of association between VIP and comparator genes’ expression data from the TCGA, GTEX, and CCLE datasets was determined using a Pearson’s correlation test (*R*). For initial screening, *R*-coefficients > |0.3| and corresponding *p*-value < 0.05 were considered statistically meaningful [30]. Gene expression data (RNALog2(norm_count+1)) for VIP and ZEB1 in healthy (GTEX) and cancer (TCGA) tissues were analyzed per a Mann–Whitney U test for non-parametric data with *p* < 0.05 defined as statistically significant using GraphPad Prism (v9.5.1).

VIP and ZEB1 Expression and Cancer Pathway Activity: Gene set analysis was performed to assess the pathway activity of VIP and ZEB1 in gastrointestinal malignancies, including COAD (colon adenocarcinoma), ESCA (esophageal carcinoma), PAAD (pancreatic adenocarcinoma), and STAD (stomach adenocarcinoma). The analysis was conducted using the GSCA (Gene Set Cancer Analysis) tool, as previously described [31], with pathway activity scores assigned in ten critical cancer-related pathways (TSC/mTOR, RTK, RAS/MAPK, PI3K/AKT, hormone ER, hormone AR, EMT, DNA damage response, cell cycle, and apoptosis) based on a total of 7876 TCGA samples across 32 different cancer types. Pathway activity was determined by stratifying samples into high and low-expression groups based on the median gene expression, and a score was then calculated using a student *t*-test, with *p*-values adjusted by the false discovery rate (FDR). Reported results were considered significant when the FDR was <0.05, indicating a low likelihood of false positives.

Estimate, Stromal, and Immune Scoring: Estimate, stromal, and immune scores were used to compare tumor purity, stromal cell presence, and immune cell infiltration in stomach adenocarcinoma samples based on VIP and ZEB1 high vs. low tumor expression using the ESTIMATE scoring system, as previously described (https://bioinformatics.mdanderson.org/estimate/index.html (accessed on 8 June 2023)) [32]. A total of 377 of the 415 STAD samples with scoring available were evaluable (nulls removed and primary tumor only). The median estimate, stromal, and immune scores were analyzed per the Mann–Whitney U test for non-parametric data with *p* < 0.05 defined as statistically significant using GraphPad Prism (v9.5.1)

## 3. Results

### 3.1. VIP Expression in Cancer

**Figure 1:**
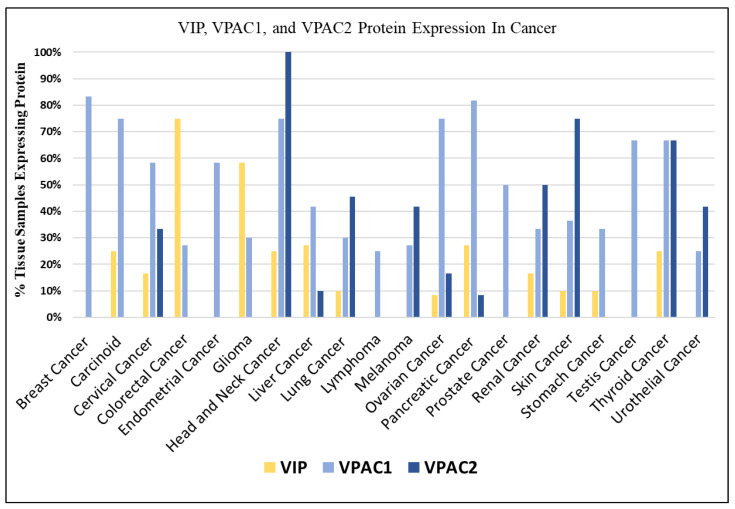
Protein expression of VIP and VIP Receptors In Cancer: VIP expression is highest in gliomas and colorectal, pancreas, thyroid cancers. VPAC1 is generally expressed on all cancer types and VPAC2 expression is limited to certain cancer types including those of the head and neck, lung, skin, kidney, thyroid, bladder, and melanoma.

The protein expression of VIP, VPAC1, and VPAC2 was investigated using immunohistochemistry data from the Human Protein Atlas (HPA) (Figure 1) [33,34]. Consistent with previous research [11], the HPA protein analysis revealed that VPAC1 is expressed in nearly all cancer histologies. In fact, VPAC1 expression was observed in over 70% of tissue samples from breast, carcinoid, head and neck, ovarian, and pancreatic cancers. On the other hand, the expression of VIP varied among different tumor histologies, with several histologies showing no detectable protein expression. However, it is noteworthy that many of the VIP non-expressing histologies still exhibited the expression of VPAC1 or VPAC2 receptors. For example, cancers of the breast, endometrium, prostate, testis, urothelium/bladder, and melanoma showed an expression of VPAC1 or VPAC2 despite lacking VIP expression. The differential expression patterns of VIP and its receptors across different histologies suggest distinct roles and potential interactions between VIP-receptor signaling and other pathways in cancer. Therefore, we undertook further investigations to elucidate the functional significance of these observations in the context of specific cancers.

**Figure 2:**
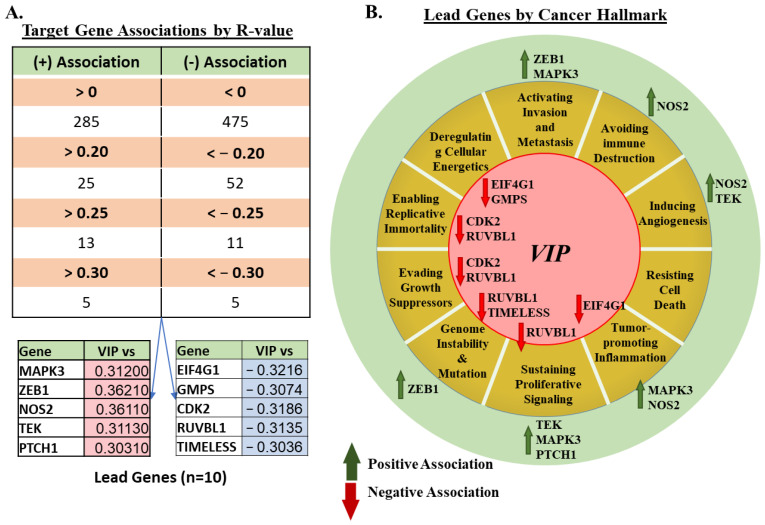
10 Lead Genes Identified and Associated Cancer Hallmarks: (**A**) 10 lead-genes identified with R values > |0.3| for the entire analyzed dataset. (**B**) Further analysis by cancer hallmark shows potential associations of VIP with hallmarks other than proliferative signaling.

To further characterize the signaling landscape, VIP mRNA expression was compared against a panel of 760 cancer-pertinent genes across all evaluable tissue samples in the TCGA PANCAN database. The selected gene panel was based on the Nanostring nCounter^®^ Tumor Signaling 360^TM^ testing panel [28]. A total of 285 genes showed positive *R* associations with VIP expression, while 475 were negatively associated (Figure 2A). Only ~10% of each cohort of positively or negatively associated genes demonstrated an *R* > |0.2| (25/285 positive correlation, 52/475 negative correlation). Based on our pre-specified cutoff of *R* > |0.3|, five positively correlated and five negatively correlated lead genes were identified (positive correlation: MAPK3, ZEB1, NOS2, TEK, and PTCH1; negative correlation: EIF4G1, GMPS, CDK2, RUVBL1, and TIMELESS) (all *p* < 0.05, Supplemental Figure S1.2).

### 3.2. mRNA Expression of VIP Associated Genes by Cancer Hallmark and Tissue Histology

To provide functional context to our findings, we also examined the association of the genes of interest with the pathways involved in the cancer hallmarks. Eight genes (CPA3, FAM30A, FOXP3, HDC, HSD11B1, PNIC, SH2D1A, and TCL1A) were excluded due to their primary involvement in the functions of non-malignant immune cells. Amongst the 752 genes remaining, the majority are involved in cancer hallmarks for activating sustaining proliferative signaling (n = 223), tumor-promoting inflammation (n = 178), invasion and metastasis (n = 146), and avoiding immune destruction (n = 122), with some overlap in certain genes across multiple cancer hallmarks (Supplemental Figure S1.3). Further focusing on lead gene functions, mapping by cancer hallmark, shows that VIP may be involved in the up- or down-regulation of genes relevant to nearly all cancer hallmarks (other than resisting cell death), with the higher number of lead gene associations seen in the hallmark pathway of “sustaining proliferative signaling” (RUVBL1, TEK, MAPK3, and PTCH1) (Figure 2B).

**Figure 3:**
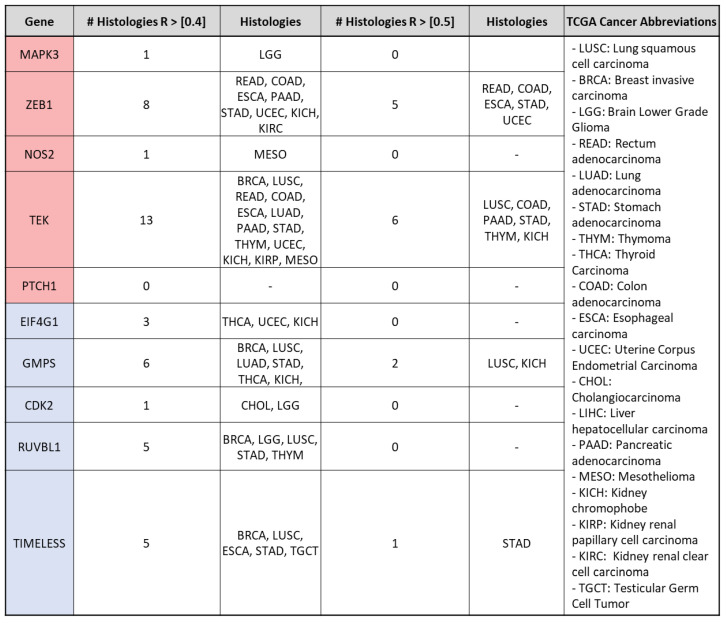
Lead Gene associations by tumor histology and increasing R-value Strength: Lead genes with corresponding histologies by increasing R-value strength. At R > |0.5|, only 4/10 lead genes retain meaningful associations with VIP.

**Figure 4:**
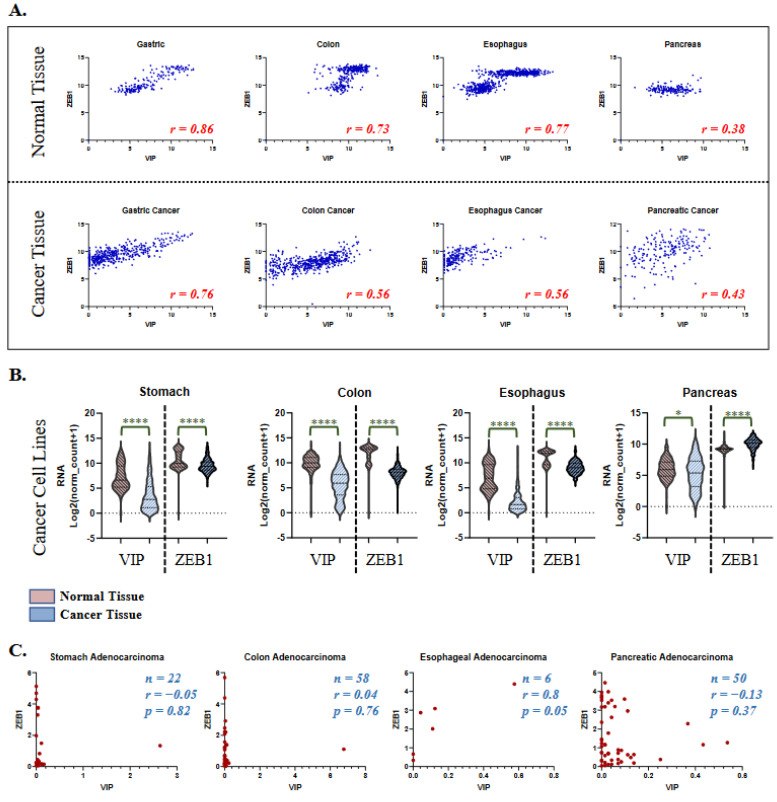
VIP and ZEB1 mRNA expression in gastrointestinal cancer and health tissues: (**A**) Similar VIP vs ZEB1 associations are seen in gastric, colon, esophageal, and pancreatic cancer (TCGA) and healthy (GTEX) tissues (*p* < 0.0001 for all analyses). (**B**) Total mRNA expression of VIP and ZEB1 is reduced in cancers of the stomach, colon, and esophagus (*p* < 0.0001 (****)), increased ZEB1 in cancer tissue (*p* < 0.0001), and similar in the pancreas for VIP *p* = 0.012 (*). (**C**) VIP and ZEB1 mRNA expression (log2(TPM+1)) in human gastric (stomach), colon, and pancreatic adenocarcinoma cancer cell lines (CCLE) does not show meaningful expression correlations. Positive correlation is seen in esophageal cell lines (*R* = 0.8, *p* = 0.05).

To explore the cancer histologies where the associations between VIP and lead genes are most significant, we employed increasingly stringent *R*-coefficient cutoffs (>|0.4| and >|0.5|). The *R*-coefficients between VIP and each lead gene were determined for all assessable samples within each TCGA cancer histology (Supplemental Figure S2). When using *R* > |0.4|, nine out of the ten lead genes exhibited histology-specific associations with VIP. Among them, TEK showed the highest number of histology-specific associations (n = 13), followed by ZEB1 (n = 8) (Figure 3). When further increasing the cutoff to *R* > |0.5|, only four out of the ten lead genes retained significant associations with VIP. These genes were ZEB1, TEK, GMPS, and TIMELESS. These associations showed histologic overlap in cancers of the gastrointestinal tract (STAD and COAD), lung (LUSC), and kidney (KICH). Notably, the strongest association observed was between VIP and ZEB1 in stomach adenocarcinoma (*R* = 0.76, Figure 4A). These findings highlight the cancer histologies where the association between the expression of VIP and lead genes is particularly meaningful, based on progressively stricter *R*-coefficient criteria. The strong association between VIP and ZEB1 in stomach adenocarcinoma warranted further investigation.

Given the known importance of VIP in embryogenesis, we also sub-grouped individual cancer histologies by germ layer of origin and looked for germ layer-specific expression associations (Supplemental Figure S3A, Supplemental Table S2). In this context, we also evaluated whether cross-reactive peptide signaling influenced our findings. The PACAP (pituitary adenylate cyclase-activating polypeptide) peptide shares 67% homology with VIP and is also able to bind to VPAC1 and VPAC2, in addition to its specific PAC1 receptor (Supplemental Figure S3B) [5,35]. Associations between lead genes and VIP or PACAP stratified by germinal layer did not indicate confounding functional overlap between VIP and PACAP. Germinal layer findings for VIP revealed tumors of ectodermal and mesodermal origin demonstrated correlations with *R* > |0.4| with non-meaningful *R* < |0.2| signals seen in PTCH1 (mesoderm) and GMPS, CDK2, RUVBL1, and TIMELESS (endoderm).

### 3.3. VIP and ZEB1 Expression in Healthy Tissue and Cancer Cell Lines

Given the compelling association between VIP and ZEB1 identified in gastrointestinal malignancies, especially of the stomach and colon, we tested VIP and ZEB1 expression in their corresponding healthy tissues. The healthy gastrointestinal tissue expression of VIP and ZEB1 was analyzed using the GTEX dataset [29]. Healthy gastric, colon, esophageal, and pancreatic tissues had *R* coefficients between VIP and ZEB1, an expression similar to those seen in TCGA cancer tissues (*p* < 0.0001 for all analyses) (Figure 4A). The biphasic distribution of VIP/ZEB1 (low) vs. VIP/ZEB1 (high) seen in the colon and esophageal tissues is thought to be related to the differences in the presence of mucosal and submucosal tissue in the samples. VIP expression in healthy pancreatic tissue is likely more susceptible to hormonal regulation of the endocrine pancreatic tissue, supporting the findings of variable VIP expression with somewhat consistent ZEB1 expression.

To determine if VIP and ZEB1 expression is up-regulated or down-regulated in cancer vs. healthy tissue, the total mRNA levels of VIP and ZEB1 between healthy and cancer tissue were compared. VIP and ZEB1 expression were both lower in stomach, colon, and esophageal cancer tissue compared to healthy tissue (*p* < 0.0001, Figure 4B). There were minimally significant differences in VIP expression between normal and pancreatic cancer tissue (*p* = 0.012) and increased ZEB1 expression in cancer tissue. We next analyzed associations between VIP and ZEB1 expression in the human stomach, colon, esophageal, and pancreatic adenocarcinoma cell lines from the CCLE (cancer cell line encyclopedia) using the DepMap resource. Interestingly, the cell line expression data did not recapitulate the VIP and ZEB1 associations seen in healthy and cancer tissues and showed only a statistically significant association between VIP and ZEB1 in a limited number of esophageal adenocarcinoma cases (*p* = 0.05) (Figure 4C).

### 3.4. Gene Set Analysis of VIP and ZEB1

**Figure 5:**
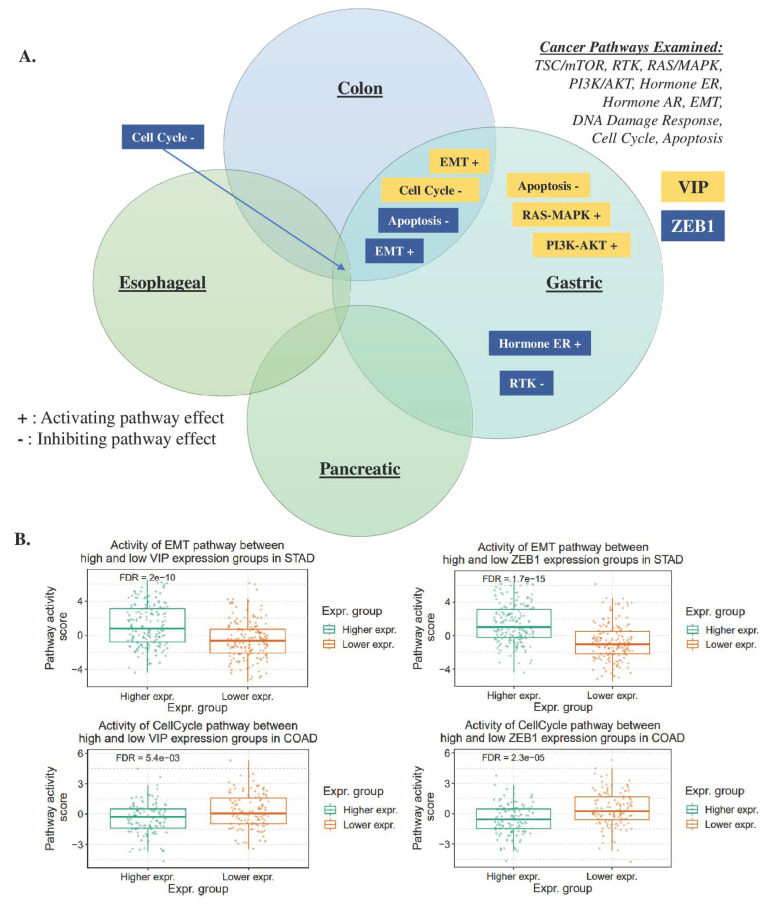
Gene Set Analysis of VIP and ZEB1 in colon, esophageal, gastric, and pancreatic cancers: (**A**) VIP and ZEB1 share activating effects on EMT and inhibitory effects on cell cycle in gastric and colon cancer. No pathway effects seen in pancreatic cancer (**B**) Representative box plots showing impact of high vs low VIP and ZEB1 expression in gastric and colon cancer in EMT activation and cell cycle inhibition (FDR < 0.25).

The consistent association between VIP and ZEB1 expression in both healthy and cancerous tissues suggests a potential linkage of the signaling pathways of these two genes. To investigate this further, gene set analysis (GSA) was conducted in colon, gastric, esophageal, and pancreatic cancers using the GSCA tool for the following cancer-related pathways: TSC/mTOR, RTK, RAS/MAPK, PI3K/AKT, hormone ER, hormone AR, EMT, DNA damage response, cell cycle, and apoptosis. GSA revealed that VIP and ZEB1 had activating effects on EMT and inhibitory effects on the cell cycle pathway in both colon and gastric cancers. Additionally, in gastric cancer, VIP and ZEB1 showed inhibitory effects on the apoptosis pathway (FDR < 0.05, Figure 5A, Supplemental Table S3). Comparisons between the high and low tissue expression of VIP and ZEB1 were performed based on the GSA results, focusing on the EMT and cell cycle pathways in gastric and colon cancer (Figure 5B). These findings indicate that VIP and ZEB1 may jointly influence the EMT and cell cycle pathways in gastric and colon cancers. Moreover, the inhibitory effects on the apoptosis pathway observed in gastric cancer suggest potential roles for VIP and ZEB1 in modulating cell survival mechanisms in this specific cancer type.

### 3.5. Estimate, Stromal, and Immune Scoring

**Figure 6:**
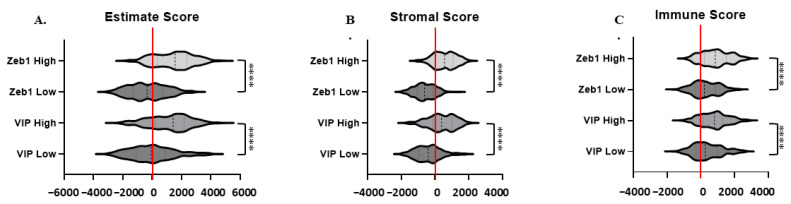
Estimate, Stromal, and Immune scores grouped by low versus high VIP and ZEB1 Expression: (**A**) Median Estimate score for ZEB1 high (1569) and ZEB1 low (−348.7) and VIP high (1428) and VIP low (−66.05) (**B**). Median Stromal score for ZEB1 high (568.2) and ZEB1 low (−621.5) and VIP high (387.2) and VIP low (−397.6) (**C**). Median Immune score for ZEB1 high (864) and ZEB1 low (215) and VIP high (814.2) and VIP low (264.1). *p* < 0.0001 for all comparisons (****).

VIP is also known to exert paracrine effects in cancer by supporting the immunosuppressive activity of CD4+CD25+ regulatory T cells and tolerogenic dendritic cells [8,36]. In vivo studies have demonstrated that treatment with VPAC peptide antagonists can reverse these immune effects and down-regulate T cell expression of the inhibitory marker PD-1, promote cytotoxic T cell differentiation and expansion, and enhance intratumoral T cell infiltration, ultimately leading to tumor elimination [15,37,38,39], and we have recently shown that inhibiting VIP in murine pancreatic cancer models improves the response to T cell checkpoint inhibitors [15]. To determine if the VIP and ZEB1 associations seen in our analysis were confounded by immune cell-mediated paracrine VIP, we compared whether high versus low VIP and ZEB1 expression correlated with tumor purity, stromal tissue presence, or immune cell infiltration using the estimate, stromal, and immune scores, respectively. ZEB1 and VIP high-expressing tumors (n = 189) were associated with higher median estimate and stromal scores, with high expression most associated with positive scores (Figure 6A,B). Median immune scores were positive for low and high ZEB1 and VIP expression, with higher scores associated with increased VIP and ZEB1 expression (Figure 6C).

## 4. Discussion

To comprehensively characterize the signaling landscape of autocrine VIP in cancer, we conducted an *in silico* gene expression analysis. Determining the downstream effects of VIP in cancer is crucial to understanding its role as a circulating biomarker and VPAC as a diagnostic, prognostic, or predictive target. The utility of radiolabeled-VIP and the development of VIP-targeted theranostics is severely limited by this lack of understanding.

Comprehensive gene expression studies of cancer are valuable when studying peptides such as VIP. The VIP sequence is highly conserved across mammals and rarely mutated in cancers, in support of a critical role for VIP-signaling in normal physiology [2]. Furthermore, protein expression studies, including techniques such as Western blot and immunohistochemistry (IHC), may not fully capture peptide expression data due to limiting factors such as the multiple cleavage steps of the prepro peptide, variability in the antibody binding, and the short half-life of the peptide.

Among the initial screening panel of 760 genes, our *in silico* model identified 10 lead genes that showed strong associations with VIP expression. Some of these genes, such as MAPK, NOS2, CDK2, and TIMELESS, have been previously associated with VIP in non-cancer models [40,41,42,43]. However, our analysis also revealed several novel associations, the most compelling being the association with the ZEB1 transcription factor.

The association between VIP and ZEB1 in gastrointestinal tissues appears to be present in both healthy and malignant tissues. ZEB1 is a well-characterized transcription factor known to play a role in the regulation of epithelial–mesenchymal transition (EMT) in cancer [44]. EMT is a process in which primary tumor cells down-regulate the expression of structural and cell adhesion molecules, such as cytokeratin and E-cadherin, and lose their epithelial markers to acquire a more aggressive, spindle cell-like mesenchymal phenotype to drive tumor metastasis and distant invasion [45].

Detecting the early signs of EMT and targeting the process pharmacologically has been a long-standing focus of cancer research, but the heterogeneity across multiple cancer histologies and redundant downstream signaling pathways involved in E-cadherin loss have limited the success of these efforts. Transcription factors critical to EMT, including ZEB1, STAT, SNAIL, and TWIST, are not ideal targets for pharmacological interventions or readily assessed through imaging techniques. Therefore, identifying upstream cell surface targets such as VPAC1 or VPAC2 could aid in the development of EMT-targeted cancer therapies [45]. Recently, Colangelo et al. uncovered an axis between TIMELESS and ZEB1 in colorectal cancers, demonstrating that the loss of TIMELESS promotes tumor progression and poor prognosis by inducing ZEB1 expression and EMT. These findings align with the negative correlation we observed between VIP and TIMELESS in our analysis [46].

However, the current study has some design limitations. The 760-gene panel used is a limited dataset, however, this selection was deliberate to focus on well-elucidated genes with known functions in cancer to facilitate the further translation of our findings. The under-representation of certain histologies in the TCGA dataset may have led to histology-specific errors and potential missed associations due to a smaller sample size. The initial screening of lead genes by R > {0.3} may appear to be a less stringent threshold, however, this was done only when evaluating the VIP-gene associations across all TCGA histologies and samples. Of note, all R in the initial screen were < {0.4}.

Findings from in silico analyses can be challenging to translate into in vitro studies or in vivo therapies. The divergent findings in VIP and ZEB1 expression associations between tissues (healthy and malignant) and cancer cell lines highlight the need for future studies in both settings. Factors such as culture conditions may affect VIP, ZEB1, and EMT-related phenotypes in vitro. Heterogeneity in the tumor microenvironment and between sequenced samples may also affect data interpretation, however, the estimate, stromal, and immune scoring data support that our findings are not confounded by the paracrine effects of immune cell infiltrates and are cancer cell and cancer stroma dependent. The differences seen by the cancer-mediated stromal cell presence may also indicate why the patterns vary between healthy tissues and cancer tissues.

The identified association between ZEB1 and VIP expression may not be causal. However, the consistent association of the expression levels for these genes across multiple cancer histologies, healthy tissues, and gene set analyses indicates potential significance and warrants further investigation. Furthermore, while the transition from a proliferative to invasive phenotype is considered fundamental to EMT, our findings that VIP is involved in both processes support more recent arguments that EMT and, conversely, MET (mesenchymal–epithelial transition) represent a continuum of states and not discrete phenotypes [47].

Taken together, the VIP–VPAC axis offers a widely expressed and targetable biomarker in cancer. The presence of VPAC in both VIP-expressing and non-expressing tissues suggests that cancers may be simultaneously susceptible to both autocrine and paracrine VIP effects. Whether targeting VPAC can be diagnostic, prognostic, and/or predictive remains to be fully explored. Our findings indicate that in gastrointestinal malignancies, further in vitro and in vivo validation is necessary to elucidate the mechanism of interaction between VIP and ZEB1 and to determine how VIP supports ZEB1-mediated EMT involved in tumor invasion and metastasis.

## 5. Conclusions

We performed hypothesis-generating in silico analyses of associations of autocrine VIP gene expression with gene targets and pathways in cancer. We identified a potential novel association of the VIP–VPAC axis with ZEB1 expression in gastrointestinal malignancies. Further studies are necessary to understand how inhibiting or activating the VIP–VPAC axis may serve as a prognostic biomarker of cancer metastasis or a predictive biomarker for response to VIP-targeted therapies on EMT in gastrointestinal malignancies.

## Data Availability

TCGA data can be accessed via UCSC Xena at (http://xena.ucsc.edu/ (accessed on 1 December 2020)). Healthy tissue data are available at the GTEX portal (https://www.gtexportal.org/home/ (accessed on 19 April 2023)). Cancer cell line data are available through the cancer cell line encyclopedia (CCCLE) available via the DepMap resource (https://depmap.org/portal (accessed on 19 April 2023)).

## Supplementary Materials

The following supporting information can be downloaded at: https://www.mdpi.com/article/10.3390/cancers15133284/s1, Supplemental Figure S1: Analyzed sample numbers, *p*-values, and pertinent cancer hallmarks for the 760 target gene panel; Supplemental Figure S2: Heat map of R-values for lead gene associations by cancer histology; Supplemental Figure S3: Heat map of lead gene associations by tissue germ layer; Supplemental Table S1: R-Values in target gene set by cancer hallmark with heat map; Supplemental Table S2. TCGA tumor histologies and abbreviations by germ layer; and Supplemental Table S3: Gene set analysis data for VIP and ZEB1.
