# Supplementary material for: Gene Expression Analysis Links Autocrine Vasoactive Intestinal Peptide and ZEB1 in Gastrointestinal Cancers
Source: Cancers (Basel). 2023 Jun 22;15(13):3284. doi: 10.3390/cancers15133284 (PMC10340472; doi:10.3390/cancers15133284)
Supplement: Supplementary file 1 [file cancers-15-03284-s001.zip › cancers-2450413-supplementary.pdf]

Supplemental Table 1. R-Values in Target-Gene Set by Cancer Hallmark with Heat Map: Positive associations denoted in red, negative in blue with strength of association based on color intensity. R-value listed in each column for which there is an associated cancer hallmark.

Gene	Activating Invasion and Metastasis	Avoiding immune Destruction	Deregulating Cellular Energetics	Enabling Replicative Immortality	Evading Growth Suppressors	Genome Instability & Mutation	Inducing Angiogenesis	Resisting Cell Death	Sustaining Proliferative Signaling	Tumor-promoting Inflammation
ABCC1	-	-	-0.1588	-	-	-	-	-	-	-
ABCC4	-	-	-	-	-	-	-	-	-0.01755	-
ABCF1	-	-	-	-	-	-	-	-	-	-
ABCG2	-	-	-	0.2541	-	-	-	-	-	-
ABI1	-	-	-	-	-	-	0.03186	-	-	-
ABL1	-	-	-	-	-	-	-	-	0.1138	-
ACKR2	-	-	-	-	-	-	-	-	-	-0.03616
ACKR3	-	-	-	-	-	-	-	-	-	0.08758
ACOT12	-	-	0.01534	-	-	-	-	-	-	-
ACSL3	-	-	-0.0651	-	-	-	-	-	-0.0651	-
ACTG2	-	-	-	-	-	-	-	-	0.1932	-
ADH1A	-	-	0.1108	-	-	-	-	-	-	-
ADM	-	-	-	-	-	-	-0.01876	-	-	-
ADORA2A	-	0.05374	-	-	-	-	-	-	-	-
ADORA2B	-	0.08245	-	-	-	-	-	-	-	-
AGO2	-	-	-	-	-	-0.1426	-	-	-0.1426	-
AGO4	-	-	-	-	-	0.05091	-	-	0.05091	-
AIMP2	-	-	-	-	-	-	-	-	-0.1091	-
AKT1	-0.13840	-	-	-	-	-	-	-	-0.13840	-
AKT1S1	-	-	-0.0811	-	-	-	-	-	-0.0811	-
AKT2	-	-	-	-	-	-	-	-	-0.1932	-
AKT3	-	-	-	-	-	-	-	-	0.1078	-
ALDH1A3	-	-	-0.06394	-	-	-	-	-	-0.06394	-
ALDOA	-	-	-0.1457	-	-	-	-0.1457	-	-	-
ALK	-	-	-	-	-	-	-	-	0.05148	-
AMOTL2	-0.03769	-	-	-	-	-	-	-	-	-
ANGPT2	-	-	-	-	-	-	0.04709	-	-	-
APC	-	-	-	-	-	-	-	-	0.1869	-
AR	-0.06652	-	-	-	-	-	-	-	-0.06652	-
ARG1	-	0.02409	-	-	-	-	-	-	-	-
ARID1A	-	-	-	-	-	-0.1399	-	-	-0.1399	-
ARID1B	-	-	-	-	-	-0.003126	-	-	-	-

ARID2	-	-	-	-	-	-0.07319	-	-	-	-
ARID4A	-	-	-	-	-	0.2553	-	-	-	-
ARID4B	-	-	-	-	-	0.03478	-	-	-	-
ASH1L	-	-	-	-	-	0.0374	-	-	-	-
ASXL2	-	-	-	-	-	-0.02925	-	-	-	-
ATF2	-	-	0.01954	-	-	0.01954	-	-	0.01954	-
ATF4	-	-	-	-	-	0.03731	-	-	0.03731	-
ATF7IP	-	-	-	-	-	-0.1245	-	-	-	-
ATG101	-	-	-0.238	-	-	-	-	-	-	-
ATG2B	-	-	0.08743	-	-	-	-	-	-	-
ATG4B	-	-	0.04174	-	-	-	-	-	-	-
ATM	-	-	-	0.09058	-	0.09058	-	-	-	-
ATOX1	-	-	-0.1337	-	-	-	-	-	-	-
ATP7A	-	-	-0.05686	-	-	-	-	-	-	-
ATR	-	-	-	-	-	-0.109	-	-	-	-
ATRX	-	-	-	-	-	0.1194	-	-	-	-
ATXN7	-	-	-	-	-	0.0344	-	-	-	-
AURKA	-0.17210	-	-0.17210	-	-0.17210	-	-	-	-	-
AURKB	-	-	-	-	-0.1897	-	-	-	-	-
AXL	0.09935	-	-	-	-	-	-	-	-	-
B2M	-	0.02963	-	-	-	-	-	-	-	0.02963
B4GALNT1	-	0.09147	-	-	-	-	-	-	-	-
B4GALT1	-	-	-	-	-	-	-	-	-0.2492	-
BAD	-	-	-	-	-	-	-	0.007956	-	-
BAX	-	-	-	-	-	-0.0731	-	-0.0731	-	-
BCAP31	-	-0.118	-	-	-	-	-	-	-	-
BCL2	-	-	-	0.001467	-	-	-	0.001467	-	-
BCL2A1	-	-	-	-	-	-0.09684	-	-0.09684	-	-0.09684
BCL2L1	-	-	-	-	-	-0.01535	-	-0.01535	-	-0.01535
BHLHE40	-	-	-0.1036	-	-	-	-	-	-0.1036	-
BIRC3	-	-	-	-	-	0.04297	-	-	-	0.04297
BIRC5	-0.20210	-	-	-	-	-	-	-	-	-
BLK	-	0.05693	-	-	-	-	-	-	-	-
BLM	-	-	-	-	-	-0.08186	-	-	-	-
BMPR1A	-	-	-	-	-	-	-	-	0.1043	-
BMPR2	-	-	-	-	-	-	-	-	0.1193	-
BRAF	-	-	-	-	-	-	-	-	-0.2096	-
BRCA1	-	-	-	-	-	-0.117	-	-	-0.117	-
BRCA2	-	-	-	-	-	-0.1006	-	-	-	-

BRIP1	-	-	-	-	-0.1851	-0.1851	-	-	-	-
BRMS1	-	-	-	-	-	-0.177	-	-	-	-
BTK	-	-	-	-	-	-	-	-	0.01493	0.01493
BUB1	-	-	-0.1995	-	-0.1995	-	-	-	-	-
BUB1B	-	-	-	-	-0.2065	-	-	-	-	-
BYSL	-	-	-	-	-	-	-	-	-0.2335	-
C3AR1	-	-	-	-	-	-	-	-	-	0.04855
CA9	-	-	-	-	-	-	0.00661	-	-	-
CACYBP	-	-	-0.09215	-	-	-	-	-	-0.09215	-
CALML3	-	-	-	-	-	-	-	-	-0.2102	-
CARD11	-	0.02141	-	-	-	-	-	-	-	0.02141
CASP3	0.00357	-	-	-	-	-	-	0.00357	-	-
CAV1	0.00096	-	-	-	-	-	-	-	-	-
CBX2	-	-	-	-	-	-0.1919	-	-	-	-
CCL13	-	-	-	-	-	-	-	-	-	-0.0303
CCL19	-	-	-	-	-	-	-	-	-	0.01663
CCL2	0.03352	-	-	-	-	-	-	-	-	0.03352
CCL21	0.09706	-	-	-	-	-	-	-	-	0.09706
CCL4	-	-	-	-	-	-	-	-	-	0.02689
CCL5	-	-	-	-	-	-	-	-	-	-0.1156
CCNA2	-	-	-	-0.1399	-0.1399	-	-	-	-0.1399	-
CCNB1	-	-	-	-	-0.2128	-	-	-	-	-
CCNB2	-	-	-	-	-0.21	-	-	-	-	-
CCND1	-	-	-	-	-0.02217	-	-	-	-	-
CCND2	-	-	-	-	0.2944	-	-	-	-	-
CCNE1	-	-	-	-0.146	-0.146	-	-	-	-0.146	-
CCNE2	-	-	-	-0.07698	-0.07698	-	-	-	-	-
CCNF	-	-	-0.1962	-	-0.1962	-	-	-	-	-
CCNK	-	-	-	-	-0.1374	-0.1374	-	-	-	-
CCNT1	-	-	-	-	-0.0007515	-0.0007515	-	-	-0.0007515	-
CCR1	-	-	-	-	-	-	-	-	-	-0.005211
CCR2	-	-	-	-	-	-	-	-	-	-0.02073
CCR4	-	-	-	-	-	-	-	-	-	-0.001629
CCR5	-	-	-	-	-	-	-	-	-	-0.05504
CCR7	-0.06209	-	-	-	-	-	-	-	-	-0.06209
CCS	-	-	-0.006657	-	-	-	-	-	-	-
CD14	-	-	-	-	-	0.008913	-	-	-	0.008913
CD163	-	-0.04781	-	-	-	-	-	-	-	-
CD19	-	-0.003669	-	-	-	-	-	-	-	-

CD209	-	0.0402	-	-	-	-	-	-	-	-
CD22	-	-0.001831	-	-	-	-	-	-	-	-
CD244	-	0.01304	-	-	-	-	-	-	-	-
CD247	-	-0.02316	-	-	-	-	-	-	-	-
CD27	-	-0.04435	-	-	-	-	-	-0.04435	-	-
CD274	-0.07549	-0.07549	-	-	-	-	-	-	-	-
CD276	-	-0.246	-	-	-	-	-	-	-	-
CD28	-	-0.0962	-	-	-	-	-	-	-	-
CD300A	-	-	-	-	-	-	-	-	-	0.03178
CD33	-	-	-	-	-	-	-	-	-	0.02649
CD34	-	-	-	0.2309	-	-	-	-	-	-
CD38	-	-0.0008862	-	-	-	-	-	-	-	-
CD3D	-	-0.06696	-	-	-	-	-	-	-	-
CD3E	-	-0.0776	-	-	-	-	-	-	-	-
CD3G	-	-0.0441	-	-	-	-	-	-	-	-
CD4	-	-0.02434	-	-	-	-	-	-	-	-
CD40	-	-0.05459	-	-	-	-0.05459	-	-	-	-0.05459
CD40LG	-	0.05196	-	-	-	-	-	-	-	0.05196
CD44	-0.05629	-0.05629	-	-0.05629	-	-	-	-	-	-0.05629
CD47	-0.03208	-	-	-	-	-	-	-	-	-0.03208
CD53	-	-	-	-	-	-	-	-	-	0.02249
CD6	-	-0.03955	-	-	-	-	-	-	-	-
CD68	-	-	-	-	-	-	-	-	-	-0.05926
CD80	-	-0.09567	-	-	-	-	-	-	-	-
CD84	-	-0.001256	-	-	-	-	-	-	-	-
CD86	-	-0.02289	-	-	-	-0.02289	-	-	-	-
CD8A	-	-0.03925	-	-	-	-	-	-	-	-
CD8B	-	-0.02905	-	-	-	-	-	-	-	-
CDC20	-0.21180	-	-	-	-0.21180	-	-	-	-	-
CDC25A	-	-	-0.1304	-	-0.1304	-	-	-	-	-
CDC25C	-	-	-	-	-0.151	-	-	-	-	-
CDCA5	-	-	-	-	-0.1835	-	-	-	-	-
CDCA8	-	-	-	-	-0.1904	-	-	-	-	-
CDCP1	-	-	-	-	-	-	-	-	-0.07795	-
CDH1	-0.12950	-	-	-	-	-	-	-	-	-
CDH11	0.11360	-	-	-	-	-	-	-	-	-
CDH5	0.23290	-	-	-	-	-	-	-	-	-
CDK1	-	-	-	-	-0.1678	-	-	-	-0.1678	-
CDK12	-	-	-	-	-	-0.1402	-	-	-	-

CDK2	-	-	-	-0.3186	-0.3186	-	-	-	-	-
CDK4	-	-0.1838	-	-0.1838	-0.1838	-	-	-	-0.1838	-
CDK6	-	-	-	0.081	0.081	-	-	-	0.081	-
CDKN1A	-	-	-	0.03326	0.03326	-	-	-	-	-
CDKN2A	-	-	-	-0.156	-0.156	-	-	-	-	-
CEACAM1	0.04636	0.04636	-	-	-	-	-	-	-	0.04636
CEACAM3	-	-	-	-	-	-	-	-	-	0.1284
CENPA	-	-	-	-	-0.198	-	-	-	-	-
CFLAR	-	-	-	-	-	-	-	-	-	-0.005696
CGAS	-	-	-	-	-	-	-	-	-	-0.07592
CHD8	-	-	-	-	-	-	-	-	-0.188	-
CHEK1	-	-	-	-	-	-0.1595	-	-	-	-
CHEK2	-	-	-	-	-	-0.2262	-	-	-	-
CHMP2A	-	-	-0.03064	-	-	-	-	-	-	-
CHUK	-	-	-	-	-	-	-	-	-	0.06036
CLOCK	-	-	-	-	-	-0.01436	-	-	-	-
CLSPN	-	-	-	-	-0.185	-0.185	-	-	-	-
CMKLR1	-	-	-	-	-	-	-	-	-	0.06922
COL4A1	0.03235	-	-	-	-	-	0.03235	-	-	-
COL4A2	0.01758	-	-	-	-	-	0.01758	-	-	-
COL6A1	-0.02195	-	-	-	-	-	-0.02195	-	-	-
COL6A2	-0.06060	-	-	-	-	-	-0.06060	-	-	-
COL6A3	-0.06082	-	-	-	-	-	-0.06082	-	-	-
COP1	-	-	-	-	-0.1278	-0.1278	-	-	-	-
COX5B	-	-	0.02887	-	-	-	-	-	-	-
CPA3	-	-	-	-	-	-	-	-	-	-
CPT2	-	-	-0.03402	-	-	-	-	-	-	-
CREB1	-	-	0.01251	-	-	-	-	-	0.01251	-
CREBBP	-	-	-	-	-	-0.03668	-	-	-	-
CRK	-	-	-	-	-	-	0.01623	-	0.01623	0.01623
CSF1R	-	-	-	-	-	0.07303	-	-	-	0.07303
CSF2RA	-	-	-	-	-	-	-	-	0.03519	0.03519
CSF2RB	-	-	-	-	-	-	-	-	0.0656	0.0656
CSF3R	-	-	-	-	-	-	-	-	-	0.033
CTAG1A/B	-	-0.1076	-	-	-	-	-	-	-	-
CTLA4	-	-0.0559	-	-	-	-	-	-	-	-
CTNNA1	-0.08949	-	-	-	-	-	-	-	-	-
CTNNB1	-0.02176	-	-	-	-	-	-	-	-0.02176	-
CTNND1	-0.04985	-	-	-	-	-	-	-	-	-

CTSW	-	-0.09058	-	-	-	-	-	-	-	-
CXCL10	-	-	-	-	-	-	-	-	-	-0.1617
CXCL11	-	-	-	-	-	-	-	-	-	-0.07826
CXCL9	-	-	-	-	-	-	-	-	-	-0.1227
CXCR2	-	-	-	-	-	-	-	-	-	0.09387
CXCR4	-0.04040	-	-0.04040	-	-	-	-	-	-	-0.04040
CXCR6	-	-	-	-	-	-	-	-	-	-0.06558
CXXC5	-	-	-	-	-	-	-	-	0.1316	-
CYBB	-	-	0.02732	-	-	-	-	-	-	0.02732
CYFIP1	-	-	-	-	-	-	-	-	-	-0.1344
CYP4A11/22	-	-	0.03954	-	-	-	-	-	-	-
DCN	0.08491	-	-	-	-	-	-	-	0.08491	-
DICER1	-	-	-	-	-	0.0149	-	-	-	-
DKC1	-	-	-	-0.1536	-0.1536	-	-	-	-	-
DLL3	-	-	-	-	-	-	-	-	-0.02917	-
DNA2	-	-	-	-0.09779	-0.09779	-0.09779	-	-	-	-
DNAJC14	-	-	-	-	-	-	-	-	-	-
DNMT1	-0.17720	-	-	-	-	-	-	-	-	-
DOCK1	0.05697	-	-	-	-	-	-	-	-	-
DOCK2	-	-	-	-	-	-	-	-	-	0.01559
DTL	-	-	-	-	-	-0.2146	-	-	-	-
DUSP6	-	-	-	-	-	-	-	-	0.05735	-
E2F1	-	-	-	-0.1596	-0.1596	-	-	-	-	-
E2F2	-	-	-	-0.1658	-0.1658	-	-	-	-	-
EDN1	-	-	-	-	-	-	0.2543	-	-	-
EGF	0.08977	-	-	-	-	-	-	-	-0.08977	-
EGFR	0.09866	-	-	-	-	-	-	-	0.09866	-
EGLN3	-	-	0.04038	-	-	-	0.04038	-	-	-
EHHADH	-	-	-0.0119	-	-	-	-	-	-	-
EIF2AK3	-	-	-0.06326	-	-	-	-	-	-	-
EIF4EBP1	-	-	-0.19	-	-	-	-	-	-0.19	-
EIF4G1	-	-	-0.3216	-	-	-	-	-	-	-0.3216
ELOVL5	-	-	-0.1252	-	-	-	-	-	-0.1252	-
EME1	-	-	-	-	-	-0.2101	-	-	-	-
ENTPD1	-	0.1994	-	-	-	-	-	-	-	-
ENTPD2	-	0.1796	-	-	-	-	-	-	-	-
EOMES	-	-0.1231	-	-	-	-	-	-	-	-
EP300	-	-	-	-	-	-0.04429	-0.04429	-	-0.04429	-
EPAS1	-	-	-	0.2205	-	-	-	-	-	-

EPC1	-	-	-	-	-	0.08205	-	-	-	-
EPCAM	0.06763	-	-	-	-	-	-	-	-	-
ERBB2	-0.12880	-	-	-	-	-	-	-	-0.12880	-
ERBB3	-	-	-	-	-	-	-	-	-0.1288	-
ERCC3	-	-	-	-	-	-	-	-	-	-
ERN1	-	-	-0.02287	-	-	-	-	-	-	-
ERO1A	-	-	-0.03372	-	-	-	-	-	-	-
ESR1	-0.22510	-	-	-	-	-	-	-	-0.22510	-
ESR2	-	-	-	-	-	-	-	-	-0.02875	-
ESRP1	-0.16480	-	-	-	-	-	-	-	-0.16480	-
ESRP2	-	-	-	-	-	-	-	-	-0.1138	-
EXO1	-	-	-	-	-0.2215	-0.2215	-	-	-	-
EZH2	-0.14580	-	-	-0.14580	-	-0.14580	-	-	-	-
FAM30A	-	-	-	-	-	-	-	-	-	-
FAM83A	-	-	-	-	-	-	-	-	-0.1911	-
FANCA	-	-	-	-	-	-0.1376	-	-	-	-
FANCD2	-	-	-	-	-	-0.2266	-	-	-	-
FANCI	-	-	-	-	-	-0.2283	-	-	-	-
FAS	-	-	-	-	-	-	-	0.01929	-	-
FASLG	-	-	-	-	-	-	-	-0.07668	-0.07668	-
FBLIM1	-0.04815	-	-	-	-	-	-	-	-	-
FBLN2	0.01668	-	-	-	-	-	-	-	0.01668	-
FBN1	0.08990	-	-	-	-	-	-	-	0.08990	-
FCAR	-	-	-	-	-	-	-	-	-	0.06637
FCGR3A/B	-	-	-	-	-	-	-	-	-	-0.04794
FCRL2	-	0.04625	-	-	-	-	-	-	-	-
FEN1	-	-	-	-0.2176	-0.2176	-0.2176	-	-	-	-
FGF7	-	-	-	-	-	-	-	-	0.1603	-
FGFBP1	-	-	-	-	-	-	-	-	-0.04972	-
FGFR1	0.02356	-	-	-	-	-	-	-	0.02356	-
FGFR2	-	-	-	-	-	-	-	-	0.04275	-
FGFR3	-	-	-	-	-	-	-	-	0.04817	-
FLT1	-	-	-	-	-	-	0.2103	-	-	-
FLT3	-	-	-	-	-	-	-	-	0.02852	-
FLT4	0.19640	-	-	-	-	-	0.19640	-	-	-
FMOD	0.00168	-	-	-	-	-	-	-	0.00168	-
FOLH1	-	-0.01619	-	-	-	-	-	-	-	-
FOS	-	0.1718	-	-	-	-	-	-	-	-
FOXA1	-0.07080	-	-	-	-	-	-	-	-0.07080	-

FOXM1	-0.19790	-	-	-	-0.19790	-	-	-	-	-
FOXP1	-	-	-	-	-	-	-	-	0.1079	-
FOXP3	-	-	-	-	-	-	-	-	-	-
FPR1	-	-	-	-	-	-	-	-	-	0.05676
FRMD6	-0.14000	-	-	-	-	-	-	-	-	-
FSTL1	-0.00455	-	-	-	-	-	-	-	-	-
FZD1	-	-	-	-	-	-	-	-	-0.09962	-
FZD4	-	-	-	-	-	-	-	-	0.09409	-
FZD7	-	-	-	-	-	-	-	-	-0.0132	-
FZD8	-	-	-	-	-	-	-	-	0.06891	-
G6PD	-	-	-	-	-	-	-	-	-	-
GABARAP	-	-	0.01869	-	-	-	-	-	-	-
GABPA	-	-	0.1275	-	-	-	-	-	-	-
GALNT3	-	-	-	-	-	-	-	-	-0.03836	-
GLUD1	-	-	0.1181	-	-	-	-	-	-	-
GLUD2	-	-	0.02904	-	-	-	-	-	-	-
GLUL	-	-	0.001829	-	-	-	-	-	-	-
GMPS	-	-	-0.3074	-	-	-	-	-	-	-
GNAI3	-	-	-	-	-	-	-	-	-0.2027	-0.2027
GNLY	-	-0.04859	-	-	-	-	-	-	-	-
GPNMB	-	-0.2557	-	-	-	-	-	-	-	-
GPT	-	-	0.216	-	-	-	-	-	-	-
GPX1	-	-	-0.1192	-	-	-	-	-	-	-
GRAP2	-	-0.0345	-	-	-	-	-	-	-	-
GRB7	-	-	-	-	-	-	-0.1186	-	-0.1186	-
GREM1	0.16780	-	-	-	-	-	-	-	0.16780	-
GRHL2	-0.07948	-	-	-	-	-	-	-	-	-
GSK3B	-0.09780	-	-	-	-	-	-	-	-0.09780	-
GSR	-	-	-0.05807	-	-	-	-	-	-0.05807	-
GSTM4	-	-	0.09789	-	-	-	-	-	-	0.09789
GTSE1	-	-	-	-	-0.175	-0.175	-	-	-	-
GUSB	-	-	-	-	-	-	-	-	-	-
GZMA	-	-0.05681	-	-	-	-	-	-	-	-
GZMB	-	-0.03567	-	-	-	-	-	-0.03567	-	-
GZMH	-	-0.05669	-	-	-	-	-	-	-	-
GZMK	-	-0.06374	-	-	-	-	-	-	-	-
H2AX	-	-	-	-	-	-0.08826	-	-	-	-
HAVCR2	-	-0.02688	-	-	-	-	-	-	-	-0.02688
HCK	-	-	-	-	-	-	-	-	-	-0.0258

HDAC1	-0.08158	-	-	-	-	-0.08158	-	-	-0.08158	-
HDC	-	-	-	-	-	-	-	-	-	-
HGF	0.14800	-	-	-	-	-	-	-	0.14800	-
HIF1A	-0.07794	-0.07794	-	-	-	-	-0.07794	-	-	-
HJURP	-	-	-	-	-0.1884	-	-	-	-	-
HK2	-	-	-0.1169	-	-	-	-0.1169	-	-0.1169	-
HLA-A	-	-0.02735	-	-	-	-	-	-	-	-0.02735
HLA-B	-	-0.04499	-	-	-	-	-	-	-	-0.04499
HLA-C	-	-0.04729	-	-	-	-	-	-	-	-0.04729
HLA-DQA1	-	-0.08726	-	-	-	-	-	-	-	-0.08726
HLA-DRB1	-	-0.07354	-	-	-	-	-	-	-	-0.07354
HLA-E	-	0.002752	-	-	-	-	-	-	-	0.002752
HMGA1	-	-	-	-0.117	-	-	-	-	-	-
HMOX1	-	-	-0.02936	-	-	-	-0.02936	-	-	-
HRAS	-	-	-	-	-	-	-0.1037	-	-0.1037	-
HSD11B1	-	-	-	-	-	-	-	-	-	-
HSPB1	-	-	-	-	-	-	-0.2293	-	-0.2293	-
HUWE1	-	-	-	-	-	-	-	-	-	-0.119
ICAM1	-	-	-	-	-	-	-	-	-	-0.1691
ICOS	-	-0.07522	-	-	-	-	-	-	-	-
IDH1	-	-	-0.02328	-	-	-	-	-	-	-
IDH2	-	-	-0.06898	-	-	-	-	-	-	-
IDO1	-	-0.1325	-	-	-	-	-	-	-	-
IDO2	-	-0.006639	-	-	-	-	-	-	-	-
IFNA1	-	-	-	-	-	-	-	-	-	-0.002904
IFNAR1	-	-	-	-	-	-	-	-	-	0.02886
IFNAR2	-	-	-	-	-	-	-	-	-	0.01322
IFNG	-	-0.1279	-	-	-	-	-	-	-	-0.1279
IFNGR2	-	-	-	-	-	-	-	-	-	-0.1032
IGF1R	-0.08999	-	-	-0.08999	-	-	-	-	-	-
IGFBP3	-	-	-	-	-	-	-0.02409	-	-	-
IKBKG	-	-	-	-	-	-	-	-	-	-0.05388
IL10	-	-0.05588	-	-	-	-	-	-	-	-0.05588
IL10RA	-	-	-	-	-	-	-	-	-	0.03658
IL11	-	-	-	-	-	-	-	-	-0.002611	-0.002611
IL12B	-	-	-	-	-	-	-	-	-	-0.07151
IL12RB1	-	-	-	-	-	-	-	-	-	0.01844
IL15	-	-	-	-	-	-	-	-	-	0.02082
IL15RA	-	-	-	-	-	-	-	-	-	0.08229

IL21R	-	-	-	-	-	-	-	-	-	-0.05754
IL22RA2	-	-	-	-	-	-	-	-	-	-0.06433
IL27RA	-	-	-	-	-	-	-	-	-	-0.07711
IL2RA	-	-	-	-	-	-	-	-	-0.07027	-0.07027
IL2RB	-	-	-	-	-	-	-	-	-0.1122	-0.1122
IL2RG	-	-	-	-	-	-	-	-	0.05955	0.05955
IL3RA	-	-	-	-	-	-	-	-	0.2465	0.2465
IL4R	-	-	-	-	-	-	-	-	-	0.02758
IL6	0.06027	-	-	0.06027	-	-	-	-	0.06027	0.06027
IL6ST	-	-	-	-	-	-	-	-	-	0.04042
IL7	-	-	-	-	-	-	-	-	-	0.1833
IL7R	-	-	-	-	-	-	-	-	-	0.004496
IL9R	-	-	-	-	-	-	-	-	-	-0.04019
INHBA	-	-	-	-	-	-	-	-	-0.01196	-
IQGAP1	-	-	-	-	-	-	-	-	-0.121	-
IRF1	-	-	-	-	-	-	-	-	-	-0.03153
IRF3	-	-	-	-	-	-	-	-	-	-0.05801
IRF4	-	-	-	-	-	-	-	-	-	-0.08875
IRF7	-	-	-	-	-	-	-	-	-	-0.1381
ITGA1	0.22650	-	-	-	-	-	-	-	-	-
ITGA11	0.04979	-	-	-	-	-	-	-	-	-
ITGA5	0.00384	-	-	-	-	-	-	-	-	-
ITGA8	0.16930	-	-	-	-	-	-	-	-	-
ITGAL	-0.03429	-	-	-	-	-	-	-	-	-0.03429
ITGAM	-0.01229	-	-	-	-	-	-	-	-	-0.01229
ITGAV	0.05529	-	-	-	-	-	-	-	0.05529	0.05529
ITGAX	-0.00669	-	-	-	-	-	-	-	-	-0.00669
ITGB1	0.00419	-	-	-	-	-	-	-	0.00419	0.00419
ITGB2	-0.05196	-	-0.05196	-	-	-	-	-	-	-0.05196
ITGB3	0.02126	-	-	-	-	-	-	-	0.02126	0.02126
ITGB5	-0.11670	-	-	-	-	-	-	-	-	-
ITGB6	-0.07348	-	-	-	-	-	-	-	-	-
ITGB8	0.03129	-	-	-	-	-	-	-	-	-
ITK	-	-0.008468	-	-	-	-	-	-	-	-0.008468
ITPR1	-	-	-	0.1062	-	-	-	-	-	-
ITPR3	-	-	-	-0.1172	-	-	-	-	-	-
JAG1	-	-	-	-	-	-	-	-	0.03258	-
JAK1	-	-	-	-	-	-	-	-	0.07012	0.07012
JAK2	0.14120	-	-	-	-	-	-	-	0.14120	0.14120

JAK3	-	-	-	-	-	-	-	-	-0.0392	-0.0392
JMJD1C	-	-	-	-	-	0.1894	-	-	-	-
JUN	0.13960	0.13960	-	-	-	-	-	-	0.13960	-
JUP	-0.15170	-	-	-	-	-	-	-	-	-
KAT2B	-	-	-	-	-	0.1643	-	-	0.1643	-
KAT6A	-	-	-	-	-	-0.01469	-	-	-	-
KAT6B	-	-	-	-	-	0.05291	-	-	-	-
KDM1A	-0.21730	-	-	-	-	-0.21730	-	-	-0.21730	-
KDM3B	-	-	-	-	-	0.04865	-	-	-	-
KDM5A	-	-	-	-	-	-0.193	-	-	-	-
KDM6A	-	-	-	-	-	-0.03397	-	-	-	-
KDR	-	-	-	-	-	-	0.1569	-	-	-
KEAP1	-	-	-0.0694	-	-	-	-	-	-	-
KIF20A	-	-	-	-	-0.245	-	-	-	-	-
KIF23	-	-	-	-	-0.2378	-	-	-	-	-
KIF2C	-	-	-	-	-0.2144	-	-	-	-	-
KIR2DL3	-	-0.05165	-	-	-	-	-	-	-	-
KIR2DL4	-	-0.08735	-	-	-	-	-	-	-	-
KIR2DS4	-	-0.05213	-	-	-	-	-	-	-	-
KIR3DL1	-	-0.01994	-	-	-	-	-	-	-	-
KIR3DL2	-	-0.07392	-	-	-	-	-	-	-	-
KIT	-	-	-	-	-	-	-	-	0.09207	-
KLF5	-	-	-	-	-	0.02046	-	-	-	-
KLK2	-0.08423	-	-	-	-	-	-	-	-0.08423	-
KLK3	-	0.005705	-	-	-	-	-	-	0.005705	-
KLK4	-	-	-	-	-	-	-	-	-0.08355	-
KLRB1	-	0.07641	-	-	-	-	-	-	-	-
KLRD1	-	0.0226	-	-	-	-	-	-	-	-
KLRG1	-	0.07778	-	-	-	-	-	-	-	-
KLRK1	-	0.06159	-	-	-	-	-	-	-	-
KMT2A	-	-	-	-	-	0.1588	-	-	-	-
KMT2D	-	-	-	-	-	0.02589	-	-	-	-
KPNA2	-	-	-	-	-	-0.2225	-	-	-0.2225	-0.2225
KRAS	0.02118	-	-	0.02118	-	-	-	-	0.02118	-
KRT16	-	-	-	-	-	-	-	-	-0.2228	-
KRT17	-	-	-	-	-	-	-	-	-0.1926	-
LAG3	-	-0.08997	-	-	-	-	-	-	-	-
LAIR1	-	-	-	-	-	-	-	-	-	-0.001052
LAMA2	0.13760	-	-	-	-	-	-	-	0.13760	-

LAMA4	0.13460	-	-	-	-	-	-	-	0.13460	-
LAMB1	-0.03778	-	-	-	-	-	-	-	-0.03778	-
LAMB3	-0.04359	-	-	-	-	-	-	-	-0.04359	-
LAMC1	-0.17490	-	-	-	-	-	-	-	-0.17490	-
LAMC2	-0.06996	-	-	-	-	-	-	-	-0.06996	-
LAMTOR1	-	-	-0.06153	-	-	-	-	-	-	-
LAMTOR2	-	-	-0.1482	-	-	-	-	-	-0.1482	-
LAMTOR4	-	-	0.008972	-	-	-	-	-	-	-
LAMTOR5	-	-	-0.09618	-	-	-	-	-	-	-
LAT	-	-0.0006109	-	-	-	-	-	-	-	-0.0006109
LATS1	0.02049	-	-	-	-	-	-	-	-	-
LCK	-	-0.0454	-	-	-	-	-	-	-	-0.0454
LCN2	0.00671	-	-	-	-	-	-	-	-	0.00671
LDHA	-	-	-0.1678	-	-	-	-0.1678	-	-0.1678	-
LIG1	-	-	-	-0.2133	-0.2133	-0.2133	-	-	-	-
LILRB2	-	-	-	-	-	-	-	-	-	-0.03205
LIN9	-	-	-	-	-0.1172	-	-	-	-	-
LMNB1	-	-	-	-	-	-	-	-0.1463	-	-
LOX	-0.06461	-	-	-	-	-	-	-	-0.06461	-
LRRC32	-	-	-	-	-	-	-	-	0.2133	-
LTA	-	-	-	-	-	-	-	-	-	-0.04076
LTB	-	-	-	-	-	-	-	-	-	-0.03606
LTBP1	0.04934	-	-	-	-	-	-	-	0.04934	-
LTBR	-	-	-	-	-	-	-0.1875	-	-	-0.1875
LY96	-	-	-	-	-	-	-	-	-	-0.05498
LYN	-	-	-	-	-	-	-	-	-	0.08105
MAFG	-	-	0.1176	-	-	-	-	-	-	-
MAGEC1	-	-0.1645	-	-	-	-	-	-	-	-
MAGEC2	-	-0.1397	-	-	-	-	-	-	-	-
MALL	-	-	-	-	-	-	-	-	0.0589	-
MAML2	-	-	-	-	-	-	-	-	0.1316	-
MAP2K1	-	-	-	-	-	-	-	-	0.04525	-
MAP2K2	-	-	-	-	-	-	-	-	-0.01419	-
MAP3K14	-	-	-	-	-	-	-	-	0.06996	0.06996
MAP3K7	-	-	-	-	-	-	-	-	-0.09118	-0.09118
MAPK1	0.05821	-	-	-	-	-	-	-	0.05821	-
MAPK13	-	-	-	-	-	-	0.001058	-	0.001058	-
MAPK14	-	-	-0.005261	-0.005261	-	-	-0.005261	-	-0.005261	-
MAPK3	0.31200	-	-	-	-	-	-	-	0.31200	0.31200

MAPK8	-	-	-	-	-	-	-	0.04849	0.04849	-
MCL1	-	-	-	-	-	-	-	-	-	-0.03805
MCM2	-	-	-0.2922	-	-0.2922	-	-	-	-	-
MCM4	-	-	-0.2089	-	-0.2089	-	-	-	-0.2089	-
MDM2	-	-	-	-0.1467	-0.1467	-0.1467	-	-	-	-
MET	-0.01353	-	-	-	-	-	-	-	-0.01353	-
MGA	-	-	-	-	-	-	-	-	-0.1124	-
MKI67	-	-	-	-	-0.1597	-	-	-	-	-
MLANA	-	-0.1479	-	-	-	-	-	-	-	-
MLH1	-	-	-	-	-	-0.0823	-	-	-	-
MLST8	-	-	-0.08724	-	-	-	-	-	-	-
MMP9	-0.18190	-	-	-	-	-	-	-	-	-
MNDA	-	-	-	-	-	-	-	-	-	0.03368
MRPL19	-	-	-	-	-	-	-	-	-	-
MS4A1	-	0.04733	-	-	-	-	-	-	-	-
MS4A2	-	0.1008	-	-	-	-	-	-	-	-
MS4A4A	-	0.04486	-	-	-	-	-	-	-	-
MSH2	-	-	-	-	-	-0.1898	-	-	-	-
MSLN	-	0.04268	-	-	-	-	-	-	-	-
MST1R	-	0.03704	-	-	-	-	-	-	-	-
MTHFD2	-	-	-0.08998	-	-	-	-	-	-	-
MTOR	-0.20230	-	-0.20230	-	-	-	-	-	-	-
MUC1	-0.05328	-	-	-	-	-	-	-	-	-
MUC16	-	-0.2043	-	-	-	-	-	-	-	-
MX1	-	-	-	-	-	-	-	-	-	-0.1134
MYB	-	-	-	-	-	-	-	-	-0.006724	-
MYBL2	-	-	-	-	-0.196	-	-	-	-	-
MYC	-0.01342	-	-	-0.01342	-	-0.01342	-	-	-0.01342	-
MYD88	-	-	-	-	-	-	-	-	-	-0.08544
MYL9	0.12700	-	-	-	-	-	-	-	-	-
NANOG	-0.03054	-	-	-0.03054	-	-	-	-	-	-
NBL1	-	-	-	-	-	-	-	-	0.04256	-
NCAPG	-	-	-	-	-0.1809	-	-	-	-	-
NCF1	-	-	-0.03367	-	-	-	-	-	-	-
NCF2	-	-	-0.05987	-	-	-	-	-	-	-
NCK1	-	-0.1103	-	-	-	-	-0.1103	-	-	-
NCOA2	-	-	-0.006575	-	-	-	-	-	-0.006575	-
NCOR1	-	-	0.06341	-	-	0.06341	-	-	0.06341	-
NCR1	-	-0.0385	-	-	-	-	-	-	-	-

NDUFA2	-	-	0.007326	-	-	-	-	-	-	-
NDUFB1	-	-	0.0266	-	-	-	-	-	-	-
NEIL3	-	-	-	-	-	-0.1791	-	-	-	-
NEK2	-	-	-	-	-0.1783	-	-	-	-	-
NF1	-	-	-	-	-	-	-	-	0.06864	-
NFAM1	-	-	-	-	-	-	-	-	-	0.0209
NFATC2	-	0.07985	-	-	-	-	-	-	-	-
NFE2L2	-	-	-0.0637	-	-	-	-	-	-	-
NFKB1	-0.09383	-	-	-0.09383	-	-	-	-	-	-0.09383
NFKB2	-	-	-	-	-	-	-	-	-	-0.1864
NKG7	-	-0.06863	-	-	-	-	-	-	-	-
NKX3-1	-	-	-	-	-	-	-	-	-0.129	-
NME1	-	-	-	-	-	-	-	-	-0.1972	-
NOS2	-	0.3611	-	-	-	-	0.3611	-	-	0.3611
NOS3	-	-	-	-	-	-	0.2365	-	0.2365	-
NOTCH3	-	-	-	-	-	-	-	-	-0.08621	-
NRAS	-	-	-	-	-	-	-0.09475	-	-0.09475	-
NRDE2	-	-	-	-	-	-	-	-	-	-
NSD1	-	-	-	-	-	-0.1091	-	-	-	-
NT5E	-	0.2384	-	-	-	-	-	-	-	-
NUDT2	-	-	0.03725	-	-	-	-	-	-	-
NUF2	-	-	-	-	-0.1448	-	-	-	-	-
OAS1	-	-	-	-	-	-	-	-	-	-0.005844
OAS2	-	-	-	-	-	-	-	-	-	-0.1553
OAS3	-	-	-	-	-	-	-	-	-	-0.1146
OAZ1	-	-	-	-	-	-	-	-	-	-
OSM	-	-	-	-	-	-	-	-	-	0.01809
OSMR	-	-	-	-	-	-	-	-	-	-0.1545
P4HA2	-	-	-	-	-	-	-0.07208	-	-	-
P4HB	-	-	-0.2358	-	-	-	-	-	-0.2358	-
PA2G4	-	-	-	-	-	-	-	-	-0.1461	-0.1461
PAK2	-0.15510	-0.15510	-	-	-	-	-	-	-0.15510	-
PARD3	-0.13740	-	-	-	-	-	-	-	-	-
PARP1	-	-	-	-	-	-0.2477	-	-	-	-0.2477
PARP2	-	-	-	-	-	-0.06316	-	-	-	-
PARP3	-	-	-	-	-	0.06725	-	-	-	-
PARVA	0.04004	-	-	-	-	-	-	-	-	-
PBRM1	-	-	-	-	-	-0.02638	-	-	-	-
PCLAF	-	-	-	-	-	-0.1976	-	-	-	-

PCNA	-	-	-	-0.1861	-0.1861	-0.1861	-	-	-	-
PDCD1	-	-0.0824	-	-	-	-	-	-	-	-
PDCD1LG2	-	-0.07065	-	-	-	-	-	-	-	-
PDGFB	-	-	-	-	-	-	0.1242	-	0.1242	-
PDGFRA	-	-	-	-	-	-	0.1989	-	0.1989	-
PDGFRB	-	-	-	-	-	-	0.1772	-	0.1772	-
PDK1	-	-	-0.0913	-	-	-	-0.0913	-	-	-
PDLIM5	-	-	-	-	-	-	-	-	0.0908	-
PDPK1	0.15430	-	-	-	-	-	-	-	-	-
PFKFB3	-	-	0.002074	-	-	-	0.002074	-	-	-
PFKP	-	-	0.04565	-	-	-	0.04565	-	-	-
PGF	0.08108	-	-	-	-	-	-	-	-	-
PGK1	-	-	-0.1515	-	-	-	-0.1515	-	-	-
PHB	-	-	-	-	-	-	-	-	-0.1245	-
PIK3CA	-0.11500	-	-	-	-	-	-0.11500	-	-0.11500	-
PIK3CB	-	-	-	-	-	-	-0.02276	-	-0.02276	-
PIK3CD	-	-	-	-	-	-	-	-	-0.151	-
PIK3R1	-	-	-	-	-	-	0.1788	-	0.1788	-
PIK3R2	-	-	-	-	-	-	-0.09264	-	-0.09264	-
PIK3R3	-	-	-	-	-	-	-	-	-0.08601	-
PIK3R4	-	-	-0.1841	-	-	-	-	-	-0.1841	-
PIK3R5	-	-	-	-	-	-	-	-	0.02775	-
PKM	-	-	-0.1854	-	-	-	-0.1854	-	-	-0.1854
PLA2G4A	-	-	-	-	-	-	-0.01827	-	-	-
PLAU	-	-	-	-	-	-	-	-	-	-0.1445
PLCB3	-	-	-	-	-	-	-	-	0.07426	-
PLCG2	-	-	-	-	-	-	-0.03938	-	-	-0.03938
PLEK2	-	-	-	-	-	-	-	-	-0.01323	-
PLK1	-	-	-0.1656	-	-0.1656	-	-	-	-0.1656	-
PMAIP1	-	-	-	-	-	-0.1381	-	-0.1381	-	-
PNOC	-	-	-	-	-	-	-	-	-	-
POLE	-	-	-	-0.205	-0.205	-0.205	-	-	-	-
POLR2A	-	-	-	-	-	-	-	-	-	-
POU5F1	0.03511	-	-	0.03511	-	-	-	-	-	-
PPAN	-	-	-	-	-	-	-	-	-0.1088	-
PRDX1	-	-	-0.2119	-	-	-	-	-	-	-
PRDX2	-	-	0.05405	-	-	-	-	-	-	-
PRDX6	-	-	-0.1985	-	-	-	-	-	-	-
PRF1	-	-0.04776	-	-	-	-	-	-	-	-

PRIM1	-	-	-	-0.129	-0.129	-	-	-	-	-
PRKACA	-	-	-0.04463	-	-	-	-	-	-0.04463	-
PRKCA	-	-	-	-	-	-	-	-	0.2754	-
PRKCB	-	-	-	-	-	-	0.1854	-	-	0.1854
PSMB10	-	0.04513	-	-	-	-	-	-	0.04513	0.04513
PSMB7	-	-0.1707	-	-	-	-	-	-	-0.1707	-
PSMB9	-	-0.01081	-	-	-	-	-	-	-0.01081	-0.01081
PSMC4	-	-	-	-	-	-	-	-	-	-
PTCH1	-	-	-	-	-	-	-	-	0.3031	-
PTCH2	-	-	-	-	-	-	-	-	0.2463	-
PTEN	0.10210	-	-	-	-	-	-	-	0.10210	-
PTGER1	-	-	-	-	-	-	-	-	-	0.1001
PTGER2	-	-	-	-	-	-	-	-	-	0.08781
PTGER3	-	-	-	-	-	-	-	-	-	0.1984
PTGER4	-	-	-	-	-	-	-	-	-	0.1639
PTGS2	0.07788	0.07788	-	-	-	-	-	-	0.07788	0.07788
PTPN6	-	-0.09491	-	-	-	-	-	-	-	-0.09491
PTPRC	-	0.02923	-	-	-	-	-	-	-	-
PUM1	-	-	-	-	-	-	-	-	-	-
RAC3	-0.23690	-	-	-	-	-	-	-	-0.23690	-
RAD51	-	-	-	-	-0.2012	-0.2012	-	-	-	-
RAD51AP1	-	-	-	-	-	-0.1615	-	-	-	-
RAD54B	-	-	-	-	-	-0.1775	-	-	-	-
RAD54L	-	-	-	-	-	-0.1904	-	-	-	-
RAF1	-0.11620	-	-	-	-	-	-	-	-0.11620	-0.11620
RASA1	-	-	-	-	-	-	0.1021	-	0.1021	-
RB1	-	-	-	0.09071	0.09071	-	-	-	0.09071	-
RB1CC1	-	-	0.03161	-	-	-	-	-	-	-
RBBP5	-	-	-	-	-	-0.1207	-	-	-0.1207	-
RBL1	-	-	-	-	-0.103	-	-	-	-0.103	-
RBX1	-	-	-	-	-0.1218	-	-0.1218	-	-0.1218	-
REL	-	-	-	-	-	-	-	-	-	-0.07882
RELB	-	-	-	-	-	-	-	-	-	-0.134
RET	-	-	-	-	-	-	-	-	0.116	-
RFC2	-	-	-	-0.2753	-0.2753	-	-	-	-	-
RFC3	-	-	-	-0.06226	-0.06226	-	-	-	-	-
RFC4	-	-	-	-0.2274	-0.2274	-	-	-	-	-
RICTOR	-	-	0.08878	-	-	-	-	-	-	-
RIMKLB	-	-	-0.1234	-	-	-	-	-	-	-

RNF111	-	-	-	-	-	-0.04097	-	-	-	-
ROCK1	0.02872	-	-	-	-	-	-	-	-	-
ROCK2	0.17850	-	-	-	-	-	-	-	-	-
RPA3	-	-	-	-0.1418	-0.1418	-0.1418	-	-	-	-
RPTOR	-	-	-0.08765	-	-	-	-	-	-	-
RRAGC	-	-	-0.1174	-	-	-	-	-	-	-
RRM2	-	-	-0.1776	-	-0.1776	-	-	-	-	-
RUNX1	-	-	-	-	-	-0.1257	-	-	-0.1257	-
RUVBL1	-	-	-	-0.3135	-0.3135	-0.3135	-	-	-0.3135	-
S100A12	-	-	-	-	-	-	-	-	-	-0.03997
SAV1	-0.08613	-	-	-	-	-	-	-	-	-
SDC1	-0.18740	-	-	-	-	-	-	-	-	-0.18740
SDHA	-	-	-	-	-	-	-	-	-	-
SELL	-	0.07512	-	-	-	-	-	-	-	0.07512
SERPINB5	-	-	-	-	-	-0.04592	-	-	-	-
SERPINE1	-0.01897	-	-	-	-	-	-0.01897	-	-	-
SETD2	-	-	-	-	-	-0.009938	-	-	-	-
SF3A1	-	-	-	-	-	-	-	-	-	-
SF3B1	-	-	-	-	-	-0.03033	-	-	-	-
SFN	-	-	-	-	-0.1275	-	-	-	-	-
SH2D1A	-	-	-	-	-	-	-	-	-	-
SH3PXD2A	-	-	-	-	-	-	-	-	0.05287	-
SHH	0.25120	-	-	-	-	-	-	-	0.25120	-
SIGLEC5	-	-	-	-	-	-	-	-	-	0.02892
SIGLEC9	-	-	-	-	-	-	-	-	-	-0.01769
SIN3A	-	-	-	-	-	-0.1906	-	-	-	-
SIRPA	-	-	-	-	-	-	-	-	-	0.03281
SKA1	-	-	-	-	-0.2036	-	-	-	-	-
SKIL	-	-	-	-	-	-	-	-	0.00637	-
SKP2	-	-	-	-	-0.0873	-	-	-	-0.0873	-
SLC1A5	-	-	-0.1863	-	-	-	-	-	-	-
SLC2A1	-	-	-0.1157	-	-	-	-0.1157	-	-	-
SLC7A5	-	-	-0.08221	-	-	-	-	-	-	-
SLIT2	0.03299	-	-	-	-	-	-	-	-	-
SLPI	-	-	-	-	-	-	-	-	-	-0.08189
SMAD7	0.14740	-	-	-	-	-	-	-	0.14740	-
SMC3	-	-	-	-	-0.02615	-	-	-	-0.02615	-
SMO	-	-	-	-	-	-	-	-	-0.164	-
SMS	-	-	-	-	-	-	-	-	-0.1677	-

SNAI1	0.11680	-	-	-	-	-	-	-	-	-
SNAI2	-0.15740	-	-	-	-	-	-	-	-	-
SOCS1	-	-	-	-	-	-	-	-	-	-0.07398
SOCS3	-	-	-	-	-	-	-	-	-	-0.04366
SOD1	-	-	-0.002858	-	-	-0.002858	-	-	-	-
SORD	-	-	-0.07059	-	-	-	-	-	-0.07059	-
SOS1	-	-	-	-	-	-	0.0008924	-	0.0008924	-
SOX2	-0.00559	-	-	-0.00559	-	-	-	-	-	-
SOX7	-	-	-	-	-	-	-	-	0.09488	-
SPDEF	-	-	-	-	-	-	-	-	-0.003232	-
SPHK2	-	-	-	-	-	-	0.139	-	-	-
SPIB	-	0.01806	-	-	-	-	-	-	-	-
SPINT1	-	-	-	-	-	-0.1668	-	-	-0.1668	-
SPINT2	-	-	-	-	-	-	-	-	-0.02861	-
SQLE	-	-	-0.08493	-	-	-	-	-	-	-
SQSTM1	-	-	-0.05427	-	-	-	-	-	-	-0.05427
SRC	0.02867	-	-	-	-	-	0.02867	-	0.02867	-
SREBF1	-	-	-0.163	-	-	-	-	-	-	-
SRM	-	-	-	-	-	-	-	-	-0.1731	-
SSRP1	-	-	-	-	-	-0.1934	-	-	-	-
STAG2	-	-	-	-	0.04348	-	-	-	0.04348	-
STAT1	-	-	-	-	-	-	-0.1918	-	-	-0.1918
STAT3	-0.12590	-	-	-0.12590	-	-	-0.12590	-	-0.12590	-0.12590
STAT4	-	-	-	-	-	-	-	-	-	0.1331
STING1	-	-	-	-	-	-	-	-	-	-0.02711
STIP1	-	-	-0.2523	-	-	-	-	-	-	-
STK11IP	-	-	-	-	-	-	-	-	-	-
STK3	-0.26360	-	-	-	-	-	-	-	-	-
STK4	0.07915	-	-	-	-	-	-	-	-	-
SUPT16H	-	-	-	-	-	-0.1669	-	-	-	-
SYK	-	-	-	-	-	-	-	-	-	0.02158
TAP1	-	-0.08267	-	-	-	-	-	-	-	-
TAP2	-	-0.06996	-	-	-	-	-	-	-	-
TAPBP	-	-0.1206	-	-	-	-	-	-	-	-
TAZ	0.08180	-	-	-	-	-	-	-	-	-
TBC1D10B	-	-	-	-	-	-	-	-	-	-
TBP	-	-	-	-	-	-	-	-	-	-
TBRG4	-	-	-	-	-	-	-	-	-0.1066	-
TBX21	-	-0.03826	-	-	-	-	-	-	-	-

TCF7	-	0.05583	-	-	-	-	-	-	0.05583	-
TCL1A	-	-	-	-	-	-	-	-	-	-
TCOF1	-	-	-	-	-	-	-	-	-0.1559	-
TEAD1	0.10630	-	-	-	-	-	-	-	-	-
TEAD2	-0.20250	-	-	-	-	-	-	-	-	-
TEK	-	-	-	-	-	-	0.3113	-	0.3113	-
TERF2IP	-	-	-	0.1814	-	-	-	-	-	-
TERT	-	-	-	-0.07316	-	-	-	-	-0.07316	-
TET2	-	-	-	-	-	0.0562	-	-	-	-
TFDP1	-	-	-	-	-0.01976	-	-	-	-0.01976	-
TFRC	-	-	-	-	-	-	-	-	-	-
TGFB1	-0.03240	-	-	-	-	-	-	-	-0.03240	-
TGFB2	0.04346	-	-	-	-	-	-	-	0.04346	-
TGFB3	-0.01319	-	-	-	-	-	-0.01319	-	-0.01319	-
TGFBR2	-	-	-	-	-	-	-	-	0.1999	-
TGM2	0.09536	-	-	-	-	-	-	-	-	-
THBS1	0.01588	-	-	-	-	-	0.01588	-	0.01588	-
THBS2	0.02482	-	-	-	-	-	0.02482	-	-	-
TIGIT	-	-0.08863	-	-	-	-	-	-	-	-
TIMELESS	-	-	-	-	-	-0.3036	-	-	-	-
TJP1	0.08539	-	-	-	-	-	-	-	-	-
TLK2	-	-	-	-	-	-	-	-	-	-
TLR3	-	-	-	-	-	-	-	-	-	0.07679
TLR4	0.27060	-	-	-	-	-	-	-	-	0.27060
TMPRSS2	-	-	-	-	-	0.08203	-	-	0.08203	-
TMUB2	-	-	-	-	-	-	-	-	-	-
TNF	-0.02426	-0.02426	-	-	-	-	-	-0.02426	-	-0.02426
TNFAIP3	-	-	-	-	-	-	-	-	-	-0.006543
TNFRSF17	-	-	-	-	-	-	-	0.007952	-	-
TNFRSF18	-	-0.1709	-	-	-	-	-	-0.1709	-	-
TNFRSF4	-	0.003598	-	-	-	-	-	0.003598	-	-
TNFRSF9	-	-0.116	-	-	-	-	-	-0.116	-	-
TNFSF11	-	-	-	-	-	-	-	0.0868	-	0.0868
TNFSF13B	-	-	-	-	-	-	-	-0.004895	-	-0.004895
TNFSF14	-	-	-	-	-	-	-	0.02908	-	0.02908
TNFSF8	-	-	-	-	-	-	-	0.01987	-	-
TNS1	0.19410	-	-	-	-	-	-	-	-	-
TNS4	-	-	-	-	-	-	-	-	-0.04117	-
TOP2A	-0.17920	-	-	-	-0.17920	-	-	-	-	-

TOX	-	0.2285	-	-	-	-	-	-	-	-
TOX2	-	0.0905	-	-	-	-	-	-	-	-
TOX3	-	0.2711	-	-	-	-	-	-	-	-
TOX4	-	-0.1031	-	-	-	-	-	-	-	-
TP53	-0.07970	-	-	-0.07970	-0.07970	-0.07970	-	-0.07970	-	-
TP73	-0.19870	-	-	-	-	-0.19870	-	-	-	-
TPD52	-	-	-	-	-	-	-	-	0.05667	-
TPI1	-	-	-0.1773	-	-	-	-0.1773	-	-	-
TPSAB1/B2	0.05919	-	-	-	-	-	-	-	-	-
TPX2	-	-	-	-	-0.1861	-	-	-	-	-
TRAF1	-	-	-	-	-	0.01964	-	-	-	0.01964
TRAF6	-	-	-	-	-	-	-	-	-	0.08951
TRAT1	-	-0.02861	-	-	-	-	-	-	-	-
TRRAP	-	-	-	-	-	-0.07998	-	-	-0.07998	-
TSPAN1	-	-	-	-	-	-	-	-	0.03988	-
TTK	-	-	-	-	-0.1949	-	-	-	-	-
TUBG1	-	-	-0.2788	-	-0.2788	-	-	-	-	-
TXN	-	-	-0.06284	-	-	-	-	-	-	-
TXN2	-	-	-0.03344	-	-	-	-	-	-	-
TXNRD2	-	-	-0.05838	-	-	-	-	-	-	-
TYROBP	-	-	-	-	-	-	-	-	-	-0.01357
UBB	-	-	-	-	-	-	-	-	-	-
UBE2C	-	-	-	-	-0.1644	-	-	-	-	-
UBE2T	-	-	-	-	-	-0.1667	-	-	-	-
UCHL5	-	-	-0.1168	-	-	-	-	-	-	-
UGDH	-	-	-	-	-	-	-	-	-0.1196	-
VAV1	-	-0.01244	-	-	-	-	-	-	-	-
VCP	-	-	-	-	-	-0.2327	-	-	-0.2327	-0.2327
VEGFA	0.07464	-	-	-	-	-	0.07464	-	-	-
VEGFB	-	-	-	-	-	-	-0.0384	-	-	-
VEGFC	-	-	-	-	-	-	-0.006611	-	-	-
VEGFD	-	-	-	-	-	-	0.1078	-	-	-
VWF	0.14670	-	-	-	-	-	-	-	0.14670	-
WASF2	-	-	-	-	-	-	-0.09708	-	-	-
WEE1	-	-	-	-	-0.1187	-	-	-	-	-
WNT5A	-0.03135	-	-	-	-	-	-	-	-0.03135	-
WRAP53	-	-	-	-0.1289	-0.1289	-	-	-	-	-
WWC1	0.00502	-	-	-	-	-	-	-	-	-
WWTR1	-0.09407	-	-	-	-	-	-	-	-	-

XBP1	-	-	-0.1373	-	-	-	-	-	-	-
XCL1/2	-	-	-	-	-	-	-	-	-	-0.16
XRCC2	-	-	-	-	-	-0.1656	-	-	-	-
YAP1	-0.08568	-	-	-	-	-	-	-	-	-
ZAP70	-	-0.006188	-	-	-	-	-	-	-	-0.006188
ZEB1	0.36210	-	-	-	-	0.36210	-	-	-	-
ZMYM2	-	-	-	-	-	0.08469	-	-	-	-

Supplemental Table 2. TCGA tumor histologies and abbreviations by germ Layer. 31 histologies included in the analysis (n=10,616).

Study Abbreviation	Study Name	Layer
HNSC	Head and Neck squamous cell carcinoma	Ectoderm
LUSC	Lung squamous cell carcinoma	Ectoderm
BRCA	Breast invasive carcinoma	Ectoderm
LGG	Brain Lower Grade Glioma	Ectoderm
GBM	Glioblastoma multiforme	Ectoderm
SKCM	Skin Cutaneous Melanoma	Ectoderm
READ	Rectum adenocarcinoma	Ectoderm
CESC	Cervical squamous cell carcinoma and endocervical adenocarcinoma	Ectoderm
UVM	Uveal Melanoma	Ectoderm
LUAD	Lung adenocarcinoma	Endoderm
STAD	Stomach adenocarcinoma	Endoderm
THYM	Thymoma	Endoderm
THCA	Thyroid carcinoma	Endoderm
COAD	Colon adenocarcinoma	Endoderm
ESCA	Esophageal carcinoma	Endoderm
BLCA	Bladder Urothelial Carcinoma	Endoderm
PRAD	Prostate adenocarcinoma	Endoderm
UCEC	Uterine Corpus Endometrial Carcinoma	Endoderm
CHOL	Cholangiocarcinoma	Endoderm
LIHC	Liver hepatocellular carcinoma	Endoderm
PAAD	Pancreatic adenocarcinoma	Endoderm
MESO	Mesothelioma	Mesoderm
SARC	Sarcoma	Mesoderm
UCS	Uterine Carcinosarcoma	Mesoderm
ACC	Adrenocortical carcinoma	Mesoderm
KICH	Kidney Chromophobe	Mesoderm
KIRC	Kidney renal clear cell carcinoma	Mesoderm
KIRP	Kidney renal papillary cell carcinoma	Mesoderm
OV	Ovarian serous cystadenocarcinoma	Mesoderm
TGCT	Testicular Germ Cell Tumors	Mesoderm
DLBC	Lymphoid Neoplasm Diffuse Large B-cell Lymphoma	Mesoderm

Supplemental Table 3. Gene set analysis data for VIP and ZEB1: Raw data including false discovery rate and pathway activity score differences VIP and ZEB1 gene expression in colon, esophageal, gastric, and pancreatic cancers performed using the Cancer Gene Set Analysis web-based tool.

cancertype	symbol	pathway	fdr	class	diff	entrez
COAD	VIP	EMT	0.041124418	Activation	1.015801808	7432
COAD	VIP	CellCycle	0.005431145	Inhibition	-0.750036032	7432
COAD	VIP	Apoptosis	0.198534273	None	-0.490376809	7432
COAD	VIP	DNADamage	0.489641781	None	-0.15228658	7432
COAD	VIP	Hormone AR	0.489641781	None	0.13483567	7432
COAD	VIP	Hormone ER	0.489641781	None	0.111521885	7432
COAD	VIP	PI3KAKT	0.489641781	None	0.298342695	7432
COAD	VIP	RASMAPK	0.902484051	None	0.047375737	7432
COAD	VIP	RTK	0.728515276	None	0.102467227	7432
COAD	VIP	TSCmTOR	0.902484051	None	-0.023567346	7432
COAD	ZEB1	EMT	6.7086E-07	Activation	2.029548788	6935
COAD	ZEB1	Apoptosis	0.00020995	Inhibition	-1.025632652	6935
COAD	ZEB1	CellCycle	0.000023439	Inhibition	-0.978292956	6935
COAD	ZEB1	DNADamage	0.068893755	None	-0.337543044	6935
COAD	ZEB1	Hormone AR	0.630411032	None	-0.080228178	6935
COAD	ZEB1	Hormone ER	0.068893755	None	0.213693968	6935
COAD	ZEB1	PI3KAKT	0.131204654	None	0.509012951	6935
COAD	ZEB1	RASMAPK	0.33657796	None	0.357846588	6935
COAD	ZEB1	RTK	0.093747687	None	0.355100765	6935
COAD	ZEB1	TSCmTOR	0.884069769	None	-0.02809347	6935
ESCA	VIP	Apoptosis	0.568623536	None	-0.246119895	7432
ESCA	VIP	CellCycle	0.243620801	None	-0.478107651	7432
ESCA	VIP	DNADamage	0.676039627	None	0.198821452	7432
ESCA	VIP	EMT	0.676039627	None	-0.32010165	7432
ESCA	VIP	Hormone AR	0.243620801	None	0.525147792	7432
ESCA	VIP	Hormone ER	0.73570063	None	0.040945327	7432
ESCA	VIP	PI3KAKT	0.568623536	None	0.30771803	7432
ESCA	VIP	RASMAPK	0.73570063	None	0.09607627	7432
ESCA	VIP	RTK	0.676039627	None	0.302798281	7432
ESCA	VIP	TSCmTOR	0.267783533	None	0.410803849	7432
ESCA	ZEB1	CellCycle	0.016635416	Inhibition	-0.685771683	6935
ESCA	ZEB1	Apoptosis	0.220300049	None	-0.367110716	6935
ESCA	ZEB1	DNADamage	0.900483421	None	0.063481325	6935
ESCA	ZEB1	EMT	0.108255466	None	1.050990534	6935
ESCA	ZEB1	Hormone AR	0.799326743	None	-0.157139211	6935
ESCA	ZEB1	Hormone ER	0.76083447	None	0.089393516	6935
ESCA	ZEB1	PI3KAKT	0.220300049	None	0.453095586	6935
ESCA	ZEB1	RASMAPK	0.900483421	None	0.031278487	6935
ESCA	ZEB1	RTK	0.108255466	None	-0.800094109	6935
ESCA	ZEB1	TSCmTOR	0.900483421	None	0.066770235	6935
PAAD	VIP	Apoptosis	0.783698173	None	-0.273138534	7432
PAAD	VIP	CellCycle	0.783698173	None	-0.183215654	7432
PAAD	VIP	DNADamage	0.783698173	None	0.161417247	7432
PAAD	VIP	EMT	0.783698173	None	-0.302568373	7432
PAAD	VIP	Hormone AR	0.783698173	None	0.14380997	7432
PAAD	VIP	Hormone ER	0.783698173	None	0.073908764	7432
PAAD	VIP	PI3KAKT	0.783698173	None	0.638949576	7432
PAAD	VIP	RASMAPK	0.943444522	None	0.017980714	7432
PAAD	VIP	RTK	0.783698173	None	0.213601021	7432
PAAD	VIP	TSCmTOR	0.783698173	None	0.298967536	7432
PAAD	ZEB1	Apoptosis	0.767948305	None	0.229699318	6935
PAAD	ZEB1	CellCycle	0.668681179	None	-0.262953482	6935
PAAD	ZEB1	DNADamage	0.933262588	None	-0.106771081	6935
PAAD	ZEB1	EMT	0.159984197	None	1.209519903	6935
PAAD	ZEB1	Hormone AR	0.933262588	None	-0.077218577	6935
PAAD	ZEB1	Hormone ER	0.668681179	None	0.165702829	6935
PAAD	ZEB1	PI3KAKT	0.979001915	None	0.010255007	6935
PAAD	ZEB1	RASMAPK	0.668681179	None	0.264022384	6935
PAAD	ZEB1	RTK	0.321866219	None	0.482193088	6935
PAAD	ZEB1	TSCmTOR	0.933262588	None	-0.059628799	6935
STAD	VIP	EMT	1.95166E-10	Activation	1.769543577	7432
STAD	VIP	PI3KAKT	0.010674536	Activation	0.64595023	7432
STAD	VIP	RASMAPK	0.007757894	Activation	0.550921412	7432
STAD	VIP	Apoptosis	0.004393318	Inhibition	-0.700193804	7432
STAD	VIP	CellCycle	0.000301407	Inhibition	-0.832568902	7432
STAD	VIP	DNADamage	0.389492026	None	-0.202436184	7432
STAD	VIP	Hormone AR	0.631558732	None	-0.096431926	7432
STAD	VIP	Hormone ER	0.06290419	None	0.13455976	7432
STAD	VIP	RTK	0.411419619	None	0.20181335	7432
STAD	VIP	TSCmTOR	0.389492026	None	0.196849044	7432
STAD	ZEB1	EMT	1.74046E-15	Activation	2.142423153	6935
STAD	ZEB1	Hormone ER	0.015774055	Activation	0.177402917	6935
STAD	ZEB1	Apoptosis	4.75551E-06	Inhibition	-1.043805273	6935
STAD	ZEB1	CellCycle	6.61062E-07	Inhibition	-1.087144241	6935
STAD	ZEB1	RTK	0.023233584	Inhibition	-0.573874408	6935
STAD	ZEB1	DNADamage	0.754802545	None	0.103483125	6935
STAD	ZEB1	Hormone AR	0.839497398	None	-0.062577629	6935
STAD	ZEB1	PI3KAKT	0.754802545	None	0.121121236	6935
STAD	ZEB1	RASMAPK	0.738573328	None	-0.144098442	6935
STAD	ZEB1	TSCmTOR	0.994105824	None	0.001446582	6935

1.1

Analyzed Samples by Histology			
(n=31)	Total	Null	Analyzed
ACC	93	13	80
BLCA	436	9	427
BRCA	1236	21	1215
CESC	312	3	309
CHOL	45	0	45
COAD	545	53	492
DLBC	48	0	48
ESCA	204	8	196
GBM	602	436	166
HNSC	604	38	566
KICH	91	0	91
KIRC	944	338	606
KIRP	352	29	323
LGG	529	0	529
LIHC	438	15	423
LUAD	641	65	576
LUSC	623	71	552
MESO	87	0	87
OV	608	296	312
PAAD	196	13	183
PRAD	566	16	550
READ	183	13	170
SARC	271	6	265
SKCM	479	6	473
STAD	511	99	412
TGCT	139	0	139
THCA	580	8	572
THYM	126	4	122
UCEC	583	28	555
UCS	57	0	57
UVM	80	0	80

1.2

VIP vs Lead Gene P-values	
**MAPK3 (mitogen-activated protein kinase 3)**	4.91E-238
**ZEB1 (zinc finger E-box binding homeobox 1)**	0
**NOS2 (nitric oxide synthase 2)**	0
**TEK (TEK receptor tyrosine kinase)**	2.28E-239
**PTCH1 (patched-1 protein)**	1.56E-228
**EIF4G1 (eukaryotic translation initiation factor 4 gamma 1)**	6.31E-256
**GMPS (guanine monophosphate synthase)**	9.29E-233
**CDK2 (cyclin-dependent kinase 2)**	1.64E-249
**RUVBL1 (RuvB like AAA ATPase 1)**	4.02E-242
**TIMELESS (tim)**	6.62E-225

1.3

	(+) Association	(-) Association	# Of Genes
Activating Invasion and Mets	70	76	146
Avoiding Immune Destruction	40	82	122
Deregulating Cellular Energetics	28	77	105
Enabling Replicative Immortality	13	35	48
Evading Growth Suppressors	5	74	79
Genome Instability and Mutation	28	83	111
Inducing Angiogenesis	30	37	67
Resisting Cell Death	10	13	23
Sustaining Proliferative Signaling	96	127	223
Tumor Promoting Inflammation	75	103	178

Supplemental Figure 1. Analyzed sample numbers, p-values, and pertinent cancer hallmarks for the 760 target gene panel.

1.1) Number of samples analyzed by tumor histology

1.2) VIP vs Lead Gene p-values,

1.3) Target-genes stratified by cancer hallmark

ECTODERM											
	All	Ectoderm	BRCA	CESC	GBM	HNSC	LGG	LUSC	READ	SKCM	UVM
MAPK3	0.3120	0.3537	0.0964	-0.0017	0.2263	0.1392	0.4552	0.2770	0.2033	-0.1715	-0.1609
ZEB1	0.3621	0.4341	0.3440	0.3611	-0.0398	0.1550	-0.1824	0.3842	0.5468	0.3226	0.1933
NOS2	0.3611	0.3186	0.1027	-0.0353	-0.0376	-0.0352	0.3182	0.0518	-0.1816	0.1315	-0.1148
TEK	0.3113	0.3336	0.4936	0.3813	0.3704	0.1800	0.3062	0.5188	0.4457	0.2351	0.1458
PTCH1	0.3031	0.4040	0.2230	0.0095	-0.0992	0.0040	-0.0869	0.0707	0.0146	-0.0923	0.2382
EIF4G1	-0.3216	-0.4268	-0.3901	-0.1558	-0.2865	-0.1893	-0.3336	-0.3456	-0.2330	-0.2772	-0.0213
GMPS	-0.3074	-0.4698	-0.4385	-0.2199	-0.3172	-0.3239	-0.2850	-0.5018	-0.1909	0.0158	-0.0620
CDK2	-0.3186	-0.4687	-0.3478	-0.2001	-0.2826	-0.1947	-0.4429	-0.3372	-0.1683	-0.2561	0.0487
RUVBL1	-0.3135	-0.3270	-0.4263	-0.1571	-0.1067	-0.2456	-0.4090	-0.4065	-0.3015	-0.0388	-0.0612
TIMELESS	-0.3036	-0.4284	-0.4488	-0.3193	-0.2292	-0.3152	-0.3093	-0.4516	-0.3431	-0.0534	-0.0042

ENDODERM														
	All	Endoderm	BLCA	CHOL	COAD	ESCA	LIHC	LUAD	PAAD	PRAD	STAD	THCA	THYM	UCEC
MAPK3	0.3120	0.3380	0.0840	-0.0316	0.1961	-0.0779	0.0403	0.0931	-0.3481	0.1184	-0.0387	0.0807	0.1247	0.0712
ZEB1	0.3621	0.3348	0.2797	0.1345	0.5624	0.5958	0.0564	0.3695	0.4272	0.2290	0.7643	0.3922	-0.0217	0.5233
NOS2	0.3611	0.3428	0.1233	-0.1647	-0.0377	-0.2033	0.0929	0.2647	0.0010	-0.0464	-0.0723	0.1557	0.1176	0.1501
TEK	0.3113	0.2600	0.3414	0.2356	0.5555	0.4071	0.1877	0.4777	0.5500	0.1593	0.5571	0.3425	0.5010	0.4318
PTCH1	0.3031	0.2879	0.1454	0.0122	0.1493	0.0856	0.0861	0.2215	0.3214	0.0207	0.2563	0.0323	0.3173	0.1815
EIF4G1	-0.3216	-0.2064	-0.2672	-0.0939	-0.2033	-0.2011	-0.1787	-0.2377	-0.1502	-0.0686	-0.3292	-0.4659	0.0418	-0.4116
GMPS	-0.3074	-0.0928	-0.2056	-0.2998	-0.2770	-0.2878	-0.1402	-0.4512	-0.1608	-0.0396	-0.4315	-0.4152	-0.3923	-0.2285
CDK2	-0.3186	-0.0563	-0.0686	-0.4479	-0.2305	-0.2869	-0.0854	-0.2531	0.0384	0.0259	-0.3158	-0.2506	-0.2479	-0.0819
RUVBL1	-0.3135	-0.1886	-0.1966	-0.3331	-0.3985	-0.3879	-0.1112	-0.2898	-0.3082	-0.0105	-0.4446	0.1271	-0.4574	-0.3865
TIMELESS	-0.3036	-0.1505	-0.0694	-0.3932	-0.3433	-0.4430	-0.1009	-0.3678	-0.1605	-0.0656	-0.5386	-0.2404	-0.3190	-0.3652

MESODERM												
	All	Mesoderm	ACC	DLBC	KICH	KIRC	KIRP	MESO	OV	SARC	TGCT	UCS
MAPK3	0.3120	0.2252	0.0519	-0.1208	0.3498	0.1308	0.0869	-0.1559	0.0297	0.1396	0.0525	0.2202
ZEB1	0.3621	0.4901	0.0255	0.0504	0.4232	0.4696	0.2750	0.3801	0.2475	-0.0212	0.1890	-0.0998
NOS2	0.3611	0.4849	0.3165	0.2317	0.3432	0.2776	0.2337	0.4952	0.2452	0.2850	0.1633	0.1646
TEK	0.3113	0.5131	0.3589	0.1020	0.5893	0.1107	0.4243	0.4955	0.2401	0.2486	-0.0536	0.2982
PTCH1	0.3031	-0.0061	-0.0768	0.0589	-0.1956	0.0015	0.0873	0.1630	-0.0347	-0.0427	-0.0070	0.2592
EIF4G1	-0.3216	-0.3779	-0.1728	-0.2419	-0.4865	-0.2194	-0.2494	-0.1347	-0.1163	0.0257	-0.1888	0.2371
GMPS	-0.3074	-0.3005	-0.0245	0.0562	-0.5384	-0.3468	-0.0905	-0.1695	-0.1779	-0.0992	-0.0794	-0.1211
CDK2	-0.3186	-0.2345	-0.1798	0.1525	0.0186	0.1549	-0.1156	-0.0387	-0.1533	-0.1246	-0.2531	0.0317
RUVBL1	-0.3135	-0.4716	0.0553	0.0502	-0.0667	-0.3439	-0.3142	-0.2208	-0.2133	0.0859	-0.3611	-0.1995
TIMELESS	-0.3036	-0.3717	-0.1704	0.0553	-0.1223	-0.1824	-0.2077	-0.1352	-0.1376	-0.0970	-0.4431	0.0721

Supplemental Figure 2. Heat Map of R-values for Lead Gene Associations by Cancer Histology: R-values for VIP vs lead genes for 31 individual cancer histologies. Results shown in conjunction R-values for entire TCGA dataset (1st data column) and germ-layer grouped results (2nd data column).

A.

VIP				
**Gene**	**All**	**Ectoderm**	**Endoderm**	**Mesoderm**
**MAPK3**	0.3120	0.3537	0.3380	0.2252
**ZEB1**	0.3621	**0.4341**	0.3348	**0.4901**
**NOS2**	0.3611	0.3186	0.3428	**0.4849**
**TEK**	0.3113	0.3336	0.2600	**0.5131**
**PTCH1**	0.3031	**0.4040**	0.2879	-0.0061
**Gene**	**All**	**Ectoderm**	**Endoderm**	**Mesoderm**
**EIF4G1**	-0.3216	-0.4268	-0.2064	-0.3779
**GMPS**	-0.3074	**-0.4698**	-0.0928	-0.3005
**CDK2**	-0.3186	**-0.4687**	-0.0563	-0.2345
**RUVBL1**	-0.3135	-0.3270	-0.1886	**-0.4716**
**TIMELESS**	-0.3036	**-0.4284**	-0.1505	-0.3717

B.

PACAP				
**Gene**	**All**	**Ectoderm**	**Endoderm**	**Mesoderm**
**MAPK3**	0.2005	0.0982	0.2167	0.2093
**ZEB1**	0.3542	0.3340	**0.4527**	0.2073
**NOS2**	0.1987	0.1741	0.1410	0.2823
**TEK**	0.3508	0.3963	0.3423	0.3360
**PTCH1**	0.1618	0.1713	0.1976	0.1865
**Gene**	**All**	**Ectoderm**	**Endoderm**	**Mesoderm**
**EIF4G1**	-0.1174	-0.1383	0.0026	-0.0989
**GMPS**	-0.1128	-0.1467	0.0295	-0.1224
**CDK2**	-0.2204	-0.3366	0.0167	-0.2019
**RUVBL1**	-0.1761	-0.1728	-0.1164	-0.1898
**TIMELESS**	-0.2003	-0.1882	-0.1139	-0.2722

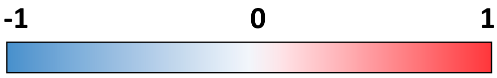

Supplemental Figure 3. Heat Map of Lead Gene Associations by Tissue Germ Layer: A) Associations of VIP with lead genes by germinal layer with focus on R > [0.4]. VIP appears to have strongest associations with lead genes in the ectodermal and mesodermal layers. **B)**

Associations of PACAP with lead genes by germinal layer. PACAP appears to have weaker associations with lead genes in the ectodermal and mesodermal layers.
